# Transglycosylation Capabilities of Wild‐Type α‐l‐Fucosidase iso1 from *Paenibacillus thiaminolyticus* and Its Engineered Mutants: Preparation of Fucosylated Oligosaccharides

**DOI:** 10.1111/1751-7915.70354

**Published:** 2026-04-23

**Authors:** Patricie Vodičková, Lucie Klimešová, Pavlína Nekvasilová, Lucie Petrásková, Helena Pelantová, Terézia Kovaľová, Petra Lipovová, Pavla Bojarová, Eva Benešová

**Affiliations:** ^1^ Department of Biochemistry and Microbiology University of Chemistry and Technology Prague Czech Republic; ^2^ Institute of Microbiology of the Czech Academy of Sciences Prague Czech Republic; ^3^ Department of Genetics and Microbiology, Faculty of Science Charles University Prague Czech Republic

**Keywords:** fucosidase, fucosyllactose, human milk oligosaccharide, *Paenibacillus thiaminolyticus*, site‐directed mutagenesis, transglycosidase, transglycosylation

## Abstract

To enhance the yield of l‐fucosylated molecules synthesized via transfucosylation, we employed α‐l‐transfucosidases, enzymes engineered from α‐l‐fucosidases to favour transglycosylation over hydrolysis. This study investigated the transglycosylation potential of mutated variants of α‐l‐fucosidase iso1 from *Paenibacillus thiaminolyticus* designed by structural comparison with *Thermotoga maritima* α‐l‐transfucosidase. Using site‐directed mutagenesis, point mutations (S237A, S237G, S237P, S237V, Y189F) were introduced, and the corresponding recombinant proteins were successfully expressed and purified. Among them, the S237V variant achieved the highest overall yield of fucosylated lactose and increased the transglycosylation/hydrolysis ratio more than 20‐fold compared to the wild‐type enzyme. This variant enabled regioselective synthesis of 3′‐and 6′‐fucosyllactose, as well as a functionalized 3′‐fucosyllactose bearing a Boc‐protected ethylthioureidyl linker (3′‐FucLac‐*t*Boc), with all structures confirmed by NMR spectroscopy. The engineered α‐l‐transfucosidase iso1 from *Paenibacillus thiaminolyticus* catalysed the regioselective synthesis of structurally defined fucosyllactose derivatives, including C‐1 functionalized analogs suitable for immobilization on biosensor surfaces or macromolecular scaffolds, thus expanding the biocatalytic toolbox and demonstrating the potential of semi‐rational enzyme design for targeted HMO‐based glycoengineering applications.

## Graphical abstract

Several point mutations were introduced into the α‐l‐fucosidase iso1 from *Paenibacillus thiaminolyticus* (α‐l‐f1*Pth*‐wt) to enhance its transfucosylation activity. The S237V variant (α‐l‐f1*Pth*‐S237V) demonstrated superior regioselectivity in the synthesis of 3′‐and 6′‐fucosyllactose and functionalized 3′‐fucosyllactose bearing a Boc‐protected ethylthioureidyl linker. The isolated products show potential in biosensor development and HMO‐based diagnostics.

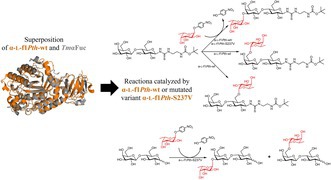

## 1 Introduction

α‐l‐Fucosylation is an essential type of glycoconjugate modification involved in many important physiological and pathological processes in living organisms. α‐l‐Fucosylated molecules are known to play key roles in growth regulation, ontogenesis, fertilization, apoptosis, inflammation, and tumour metastasis (Becker and Lowe 2003; Ma et al. 2006; Staudacher et al. 1999). Consequently, α‐l‐fucosylated molecules and their biological properties have become the focus of intense research interest, as these molecules are widely applicable, e.g., in medicine and in the food industry. However, a tailored synthesis of α‐l‐fucose‐containing compounds is not without obstacles (Miyoshi et al. 2012; Wan et al. 2020).

Two main methods are used for the preparation of α‐l‐fucosylated compounds. The commonly used chemical synthesis has two principal disadvantages: (i) low yields (e.g., Agoston et al. (2019) synthesized 2′‐fucosyllactose in an overall yield of 27%, while Pereira and McDonald (2012) reported overall yields below 20% for 2′‐fucosyllactose and 34% for 3′‐fucosyllactose) and (ii) the number of complicated and, thus, time‐consuming steps needed to perform reactions with the required stereospecificity and regioselectivity, resulting in a low overall product yield. In contrast, enzymatic synthesis has several advantages. Enzyme‐catalysed reactions are typically highly stereospecific and often exhibit significant regiospecificity. In addition, enzymatic reactions can take place under moderate conditions. Based on these parameters, the application of enzyme biocatalysts seems to be the method of choice (Bojarová and Křen 2009; Wang and Huang 2009).

Two groups of naturally occurring enzymes can be employed to produce α‐l‐fucosylated molecules. Fucosyltransferases (EC 2.4.1.) catalyse the transfer of the l‐fucosyl moiety from an activated donor to a hydroxyl group of a suitable acceptor. Although they often display high stereo‐ and regiospecificity, their nucleotide donor substrates are very specific and expensive (Hancock et al. 2006; Wang and Huang 2009; Watt et al. 1997). The other group, α‐l‐fucosidases (EC 3.2.1.51), belong to hydrolytic enzymes and thus they naturally catalyse the hydrolytic cleavage of l‐fucose from a wide range of substrates. However, under specific in vitro conditions, some of these enzymes can catalyse the synthesis of fucosylated products by transfucosylation. Unfortunately, reaction yields are considerably reduced by the simultaneous occurrence of hydrolytic activity, and despite efforts to optimize reaction conditions, low yields are often encountered (Benešová et al. 2013; Berteau et al. 2004).

Consequently, to improve transfucosylation abilities, researchers have begun to develop mutant variants of α‐l‐fucosidases. The most recent group of engineered α‐l‐fucosidases comprises the so‐called α‐l‐transfucosidases, whose transfucosylation activity is increased at the expense of their hydrolytic activity. This property is achieved by substituting one or more amino acids in their sequence, with the change usually located close to the active site. Since predicting the positions of these amino acids is complicated, several approaches have been tested.

The first approach, random mutagenesis based on directed evolution, was successfully applied in the case of α‐l‐fucosidase from *Thermotoga maritima* (*Tm*αFuc; Osanjo et al. 2007). The authors identified three mutations that resulted in significantly improved transglycosylation properties of the mutant α‐l‐fucosidase. However, considering the large number of mutants that need to be screened to identify the most effective ones, this method can be quite time‐consuming. A semi‐rational approach involves the use of a successfully engineered transfucosidase with validated mutations as a template for designing mutations in a different enzyme, as demonstrated by Saumonneau et al. (2016). Using structural alignment, they successfully identified amino acid residues in α‐l‐fucosidase from *Bifidobacterium longum* subsp. *infantis* (*Bi*AfcB) corresponding to those of α‐l‐transfucosidase from *Thermotoga maritima* , which markedly enhanced its transfucosidase activity. Several *BiAfcB* mutants were prepared by site‐directed mutagenesis, resulting in an improved transglycosylation/hydrolysis ratio, and then used for synthesis of various fucosylated human milk oligosaccharides. In the study by Zeuner et al. (2020), another structure‐guided mutagenesis strategy was applied to α‐l‐fucosidase from *Fusarium graminearum*, based on earlier findings by Osanjo et al. (2007) and Saumonneau et al. (2016). In this case, the introduced mutations mainly enhanced the enzyme's regioselectivity, rather than significantly increasing the overall transglycosylation yields.

Using a different approach, rational design, it is possible to apply molecular modelling techniques either to a known three‐dimensional structure or to a homology model developed for a yet unresolved enzyme structure with the aim of predicting the positions of amino acids with the direct influence on transglycosylation/hydrolysis activity ratio. Based on sequence alignment with selected related fucosidases, Li et al. (2017) designed several mutant variants of 1,6‐α‐l‐fucosidase from *Lactobacillus casei* (AlfC) by mutating both predicted catalytic amino acids. Their aim was to prepare fucosynthases and fucoligases, other groups of engineered fucosidases that utilize activated fucosyl donors, such as fucosyl fluorides, in synthetic glycosylation reactions (Wada et al. 2008). AlfC was also previously reported to exhibit transglycosylation activity (Becerra et al. 2015; Rodríguez‐Díaz et al. 2013). Following the elucidation of its crystal structure, Klontz et al. (2020) conducted an in‐depth investigation of its transglycosylation mechanism, employing mutational analysis, hydrogen‐deuterium exchange mass spectrometry, and molecular dynamics simulations. Interestingly, both Klontz et al. (2020) and Osanjo et al. (2007) identified mutations in structurally analogous regions of AlfC and *Tm*αFuc, suggesting a shared mechanistic basis for enhancing transfucosylation activity. These findings offer a promising path for the rational development of transfucosidase variants across different α‐l‐fucosidases. Nevertheless, no general principles have been identified yet, and the process remains unique to each enzyme. Moreover, accurate in silico modelling of such effects remains a considerable challenge.

Human milk oligosaccharides (HMOs) are among the most important biologically active fucosylated compounds. In addition to their well‐established role as prebiotics that modulate gut microbiota, HMOs also contribute to the maturation of the gastrointestinal immune system and support early neurodevelopment (Yu et al. 2025). As a result, numerous research programs have focused on exploring their synthetic production. The two predominant structures, 2′‐fucosyllactose (2′‐FL) and 3‐fucosyllactose (3‐FL), have become central to scientific interest due to their functional relevance and potential applications (Yu et al. 2025). The enzymatic synthesis of fucosyllactose by α‐l‐fucosidases is based on their ability to transfer l‐fucose from an activated donor to lactose, which serves as an acceptor. A well‐studied example is *Tm*αFuc, which utilizes *p*‐nitrophenyl α‐l‐fucopyranoside (*p*NP‐α‐l‐Fuc) as a donor and lactose as an acceptor to generate 2′‐FL (Osanjo et al. 2007). The reported conversions ranged from 25% at high substrate concentrations to 33% at very high acceptor/donor ratios (Guzmán‐Rodríguez et al. 2018a). Yields were improved up to threefold by the addition of inorganic salts (Guzmán‐Rodríguez et al. 2018b), or through enzyme immobilization achieving comparable yields even at a hundred times lower acceptor/donor ratio (Guzmán‐Rodríguez et al. 2021). Similarly, metagenome‐derived GH29 α‐l‐fucosidases, such as Fuc2358 and Fuc5372, utilize *p*NP‐α‐l‐Fuc as a donor in transglycosylation reactions with lactose. Fuc2358 specifically produced 2′‐FL and 3′‐FL regioisomers with isolated yields of 33% and 9%, respectively (Moya‐Gonzálvez et al. 2024). Furthermore, site‐directed mutagenesis led to the development of several mutant variants, which exhibited significantly higher 2′‐FL yields relative to the amount of hydrolyzed donor, demonstrating enhanced transfucosylation efficiency. Shi et al. (2020) described a novel α‐l‐fucosidase from *Pedobacter* sp. CAU209 that synthesized a mixture of 2′‐FL and 3′‐FL regioisomers, achieving a total conversion rate of up to 85%. Even more promising results were obtained with marine α‐l‐fucosidase from *Flavobacterium algicola* (OUC‐Jdch16), as only a single product was obtained with an isolated yield of up to 92% of 2′‐FL under optimized conditions (Zhou et al. 2022). Overall, α‐l‐fucosidase‐catalysed synthesis is typically donor‐limited and provides modest conversion yields, although enzyme source, reaction conditions, and immobilization strategies can significantly influence performance in specific cases.

In this work, we investigate the synthetic potential of α‐l‐fucosidase iso1 from *Paenibacillus thiaminolyticus* and its engineered variants with reduced hydrolytic activity. Point mutations were introduced using site‐directed mutagenesis, guided by structural comparison with α‐l‐transfucosidase from *Thermotoga maritima* (Osanjo et al. 2007). The transfucosylation efficiency was evaluated for both the wild‐type enzyme and its mutant variants. Based on these findings, we successfully applied the most promising variant for the regioselective synthesis of fucosylated lactose derivatives, including production on a preparative scale.

## 2 Experimental Procedures

### 2.1 Design of Mutations

The structure of α‐l‐fucosidase from *Thermotoga maritima* (*Tm*αFuc; PDB ID: 1HL8) was compared with the structure of α‐l‐fucosidase iso1 from *Paenibacillus thiaminolyticus* (α‐l‐f1*Pth*‐wt; PDB ID: 6GN6) using Coot molecular graphics application (Emsley et al. 2010). Point mutations in the sequence of α‐l‐f1*Pth*‐wt (FN869117) were designed based on highly active *Tm*αFuc transglycosylation mutants (T264A, Y267F, and L322P). As a result, five mutant variants were prepared: S237A, S237G, S237V, L297P, and Y189F (Supporting Information, Figure S2).

### 2.2 Site‐Directed Mutagenesis Using EMILI

The designed mutants were prepared by efficient mutagenesis independent of ligation (EMILI) as described by Füzik et al. (2014). PCR was performed using specific primers with the desired mutations (Supporting Information, Table S1). As a template for PCR, we used the expression construct pET16b‐α‐l‐f1*Pth*‐wt including the gene encoding α‐l‐fucosidase iso1 from *Paenibacillus thiaminolyticus* prepared previously by us (Benešová et al. 2013).

The PCR products were then purified with the commercial QIAquick PCR Purification Kit (Qiagen GmbH, Germany) and treated with DpnI (NEB, USA) for 2 h at 37°C to remove the methylated plasmid template. To generate “sticky” self‐annealing ends, PCR products (i.e., linearized plasmids) were incubated with T4 DNA polymerase with 3′ → 5′ exonuclease activity (NEB, USA) for 5 min at 22°C. Finally, both enzymes were heat inactivated (72°C for 20 min). Competent cells *E. coli* DH5α (Sigma‐Aldrich, USA) were transformed (using the heat shock method) by few microliters of reaction mixtures and cultivated on agar plates containing Luria‐Bertani medium with 0.1 mg ml^−1^ ampicillin (LBA) at 37°C overnight. The remaining gaps inside the mutant plasmids, created after spontaneous recirculation, were repaired inside the transformed bacterial cells. Subsequently, the plasmid DNA was isolated using the GenElute HP Plasmid Midiprep Kit (Sigma‐Aldrich, USA). The isolated plasmids were analysed by agarose electrophoresis using 1% gel and their concentrations were measured spectrophotometrically using NanoDrop 1000 (Thermo Fisher Scientific, USA). The presence of inserted mutations in this newly arised production vectors was confirmed by sequencing (GATC Biotech, Germany).

### 2.3 Production of Recombinant α‐l‐f1*Pth* ‐wt and Its Mutant Variants

For the production of recombinant α‐l‐f1*Pth*‐wt and its mutant variants, each fused with a polyhistidine‐tag, competent cells of *E. coli* BL21 (DE3) (Merck, Germany) were transformed by pET16b‐α‐l‐f1*Pth*‐wt or the respective mutant plasmid using the heat shock method and grown on LBA plates at 37°C overnight. Approximately 200 grown colonies were transferred into one litre of LBA medium and cultivated at 37°C overnight at 130 rpm. The following day, all cells were harvested by centrifugation (10 min, 3700 *g*, 4°C). The obtained bacterial pellets were resuspended in 17 mL of 25 mM EPPS buffer pH 8.0 and disintegrated by the high‐pressure ONE SHOT cell disruptor (Constant Systems Limited, UK) at 1.35 kBar. Insoluble parts of the cell lysate were removed by centrifugation (20 min, 20,000 *g*, 4°C) and the resulting supernatant was used for purification. The presence of the recombinant protein was confirmed by SDS‐PAGE (Supporting Information, Figure S3).

The cell pellet with the insoluble L297P mutant was resuspended in 6 mL of 25 mM EPPS buffer pH 8.0 (Sigma‐Aldrich, USA). Two detergents, Triton X‐100 and sodium salt of *N*‐lauroylsarcosine, were tested, each at a final concentration of 1% (*w/v*), to release the insoluble protein. After 1 h of incubation at room temperature under constant stirring, the mixtures were centrifuged (20 min, 20,000 *g*, 4°C), and the resulting pellets and supernatants containing the soluble portion of the recombinant protein were analysed by SDS‐PAGE.

### 2.4 Purification of Recombinant α‐l‐f1*Pth* ‐wt and Its Mutant Variants

The supernatant containing soluble proteins was loaded onto a Ni‐NTA agarose (Qiagen GmbH, Germany) column pre‐equilibrated with 25 mM EPPS buffer pH 8.0 with 150 mM KCl (Penta, CZ) and 10 mM imidazole (Sigma‐Aldrich, USA). To remove weakly bound proteins, the column was washed with the same buffer containing 40 mM imidazole. Finally, the same phosphate buffer with 250 mM imidazole was used to elute recombinant α‐l‐f1*Pth*‐wt or the respective mutant variant interacting with the nickel ions of Ni‐NTA agarose through a polyhistidine tag. To eliminate the remaining imidazole, combined fractions displaying the desired α‐l‐fucosidase activity were loaded onto a PD10 desalting column (GE Healthcare, USA) and the enzymes were eluted with 25 mM EPPS buffer pH 8.0. The purity of recombinant proteins was determined by SDS‐PAGE, which was performed in 10% running gel under reducing conditions using dithiothreitol (Sigma‐Aldrich, USA). The separated proteins were visualized by Coomassie Brilliant Blue R250 (Sigma‐Aldrich, USA).

The concentration of purified enzymes was determined using NanoDrop 1000 (Thermo Fisher Scientific, USA). For this purpose, their molar extinct coefficients at 280 nm were calculated by ProtParam (https://web.expasy.org/protparam/); Δ*ε* = 99,030 L mol^−1^ cm^−1^ for the mutant variant Y189F and Δ*ε* = 100,520 L mol^−1^ cm^−1^ for α‐l‐f1*Pth*‐wt and the remaining mutant variants.

### 2.5 Hydrolytic Activity Assay

The hydrolytic activities of α‐l‐f1*Pth*‐wt and its five mutant variants were measured using the chromogenic substrate *p‐*nitrophenyl α‐l‐fucopyranoside (*p*NP‐α‐l‐Fuc; Biosynth AG, UK). The reaction mixtures of a total volume of 110 μL contained 100 μL of 10 mM *p*NP‐α‐l‐Fuc dissolved in 25 mM EPPS buffer pH 8.0 and 10 μL of the enzyme (the final concentration of each was 0.47 mg mL^−1^). Reactions were performed at 37°C for 10 min. 100 μL of 10% (*w/v*) Na_2_CO_3_ (Penta, CZ) was added to stop the hydrolytic reaction. The amount of released *p*‐nitrophenol was determined as the increase in absorbance at 405 nm using the calibration curve constructed for a set of calibration standards with concentrations ranged from 0 to 200 μmol L^−1^. 1 U was defined as the amount of enzyme that catalyses the release of 1 μmol of *p*‐nitrophenol per 1 min.

### 2.6 Screening of Transglycosylation Activity

The donor substrate *p*NP‐α‐l‐Fuc was first dissolved in *N*,*N*‐dimethylformamide (DMF; Lachema, CZ) in a concentration of 0.5 M due to its low solubility in aqueous solvents. Subsequently, the stock solution was added to the reaction mixture at the final concentration of 50 mmol L^−1^ of *p*NP‐α‐l‐Fuc and the final concentration of 10% DMF (*v*/*v*). The α‐l‐f1*Pth*‐wt enzyme was shown to maintain good activity in the presence of DMF (Supporting Information, Figure S4). As an acceptor of the l‐fucosyl moiety, lactose was used at the final concentration of 300 mmol L^−1^. All reaction mixtures contained α‐l‐f1*Pth*‐wt or one of the mutant variants (S237P, S237V or Y189F) at the final concentration of 0.47 mg mL^−1^. The reactions were performed in the presence of 25 mM EPPS buffer pH 8.0 at 45°C. To stop the reaction, 100 μL of 10% Na_2_CO_3_ (*w*/*v*) were added into 100 μL reaction mixture. Finally, 20 μL of 55 mM sorbitol were added to all mixtures to be used as an internal standard to quantify the conversion to fucosylated lactose by HPLC.

All samples were filtered using the sterile 0.2 μm Puradisc PVDF syringe filters (Whatman, Cytiva, USA) prior to being applied to a column. High‐speed HPLC was performed using the Prominence UFLC chromatographic system (Shimadzu, Japan) with the Supelcogel Ca column (30 cm × 7.8 mm, Sigma‐Aldrich, USA) and the Supelguard Ca pre‐column (5 cm × 4.6 mm, Sigma‐Aldrich, USA). Degassed distilled water filtered through 0.22 μm nylon membrane filters (Whatman, Cytiva, USA) was used as a mobile phase. Separation was carried out at 80°C and at a pressure approximately 3.6 MPa with a flow rate of 1.0 mL min^−1^. The samples were loaded onto the column in a volume of 10 μL. To detect all present compounds, the UV/VIS detector (Shimadzu, Japan) at 280 nm was used together with the evaporating light scattering detector (ELSD; Shimadzu, Japan) at 60°C at a pressure approximately 310 kPa. The obtained data were processed by LCsolution software (Shimadzu, Japan). The maximum conversion to fucosylated lactose was determined using a calibration curve constructed for a set of maltotriose standards (Sigma‐Aldrich, USA) in a concentration range of 0 to 8 mmol L^−1^. Transglycosylation/hydrolysis ratios were calculated by dividing the maximum concentration of fucosylated lactose (measured at the specified reaction time corresponding to the highest overall yield) by the average hydrolytic activities of the respective enzyme, as determined from previous calculations.

### 2.7 Characterization of Kinetic Properties

To determine the kinetic parameters of α‐l‐f1*Pth*‐wt and α‐l‐f1*Pth*‐S237V, *p*NP‐α‐l‐Fuc was used as a substrate within a concentration range of 0.5 to 20 mmol L^−1^. All reactions were performed analogously to the hydrolytic activity assay. Calculations of the kinetic parameters of both enzymes were performed using GraphPad Prism 9.5.1 (GraphPad Software, USA).

To calculate the kinetic parameters *K*_M_, *K*_*S*_, and *V*_max_ of α‐l‐f1*Pth*‐wt, a modified Michaelis–Menten equation including substrate inhibition was used:

$$v_{0}=\frac{V_{\max}\cdot S}{K_{M}+S+\frac{S^{2}}{K_{S}}}$$

where *v*_0_ is the initial reaction rate, *V*_max_ is the limiting reaction rate, *S* is the substrate concentration, *K*_M_ is the Michaelis constant, and *K*_*S*_ is the inhibition constant (Copeland, 2000).

To calculate the kinetic parameters *K*_M_ and *V*_max_ of α‐l‐f1*Pth*‐S237V, two different equations were used. The first was the standard Michaelis–Menten equation:

$$v_{0}=\frac{V_{\max}\cdot S}{K_{M}+S}$$

where *v*_0_ is the initial reaction rate, *V*_max_ is the limiting reaction rate, *S* is the substrate concentration, and *K*_M_ is the Michaelis constant.

The second, derived from the standard Michaelis–Menten equation, is suitable for describing simultaneous hydrolysis and transglycosylation (Nguyen et al. 2014):

$$v_{0}=\frac{\left(V_{\max1}\cdot S\right)+\left(V_{\max2}\cdot S^{2}\right)}{K_{M1}+S+\frac{S^{2}}{K_{M2}}}$$

where *v*_0_ is the initial reaction rate, *S* is the substrate concentration, *V*_max1_ is the limiting reaction rate, *K*_M1_ is the Michaelis constant (both for hydrolysis) while *V*_max2_ together with *K*_M2_ are analogous parameters for transglycosylation.

### 2.8 Analytical Transglycosylation Reactions

The optimal set‐up for preparative scale reactions was initially tested on analytical scale reactions (100 μL) and screened by TLC (TLC Sillica gel 60 F_254_, Merck, USA; *i*PrOH:H_2_O:NH_4_OH *aq*., 7:2:1 *v/v/v*), and HPLC (under the conditions detailed below). The donor/acceptor ratio and the activity of α‐l‐f1*Pth*‐wt and α‐l‐f1*Pth*‐S237V were optimized. All reactions contained 30 mM donor *p*NP‐α‐l‐Fuc, 60–300 mM acceptor, and various enzyme activities (0.53–1.06 mU mL^−1^). UV‐active compounds, such as *p*NP‐α‐l‐Fuc and Lac‐*t*Boc, were detected by TLC using UV light (254 nm). The remaining compounds were detected using sulfuric acid (5% *v/v*, EtOH) followed by carbonization at 250°C.

Analyses of analytical transglycosylation reactions were performed on a Shimadzu Prominence LC system, consisting of a CBM‐20A system controller, CTO‐10AS column oven, LC‐20 ad binary pump, SIL‐20ACHT refrigerated autosampler, SPD‐20MA diode array detector (Shimadzu, Japan), and a PL 1000 ELSD detector (Polymer Laboratories, USA). Chromatographic separation of non‐functionalized carbohydrates (lactose acceptor) was performed using a Luna NH₂ column (150 × 4.6 mm, 5 μm) preceded by a Security Guard NH₂ cartridge (4 × 3 mm, Phenomenex, USA). The mobile phase consisted of acetonitrile and water (4:1, *v/v*), with a gradient elution program as follows: (A = acetonitrile, B = water), 0 min: 21% B, 0–8 min: 21%–30% B, 8–12 min: 30%–50% B, 12–13 min: 50%–21% B, 13–15 min: 21% B (column re‐equilibration). The flow rate was set to 1.1 mL min^−1^, with the column kept at 25°C. The injection volume was 1 μL. UV detection was carried out at 200 nm with a sampling rate of 40 Hz. Evaporative light scattering detection was performed under the following conditions: nitrogen gas flow rate of 0.8 mL min^−1^, nebulizer temperature of 40°C, evaporator temperature of 85°C, and a sampling rate of 2 Hz.

The C‐1 functionalized carbohydrates, carrying the (*tert*‐butoxycarbonylamino)ethylthioureidyl (*t*Boc) linker at C‐1, were analysed using a TSKgel Amide‐80 column (250 × 4.6 mm, 5 μm), preceded by a TSKgel Amide‐80 Guardgel (3.2 × 15 mm; Tosoh, Japan). The mobile phase consisted of acetonitrile and water (4:1, *v*/*v*), with the following gradient elution profile (solvent A = acetonitrile, solvent B = water): 0–7 min: 22% B, 7–16 min: linear increase to 31% B, 16–17 min: decrease to 22% B, 17–22 min: hold at 22% B to re‐equilibrate the column. The flow rate was kept at 1.0 mL min^−1^, the column temperature was set to 25°C, and an injection volume of 1 μL. Detection was carried out at 200 nm. The obtained data were processed by LCsolution software (Shimadzu, Japan).

*Preparative Transglycosylation Reaction:* (*tert‐butoxycarbonylamido*)*ethylthioureidyl α‐l‐fucopyranosyl‐*(*1 → 3/6*′*/6*)*‐β‐d‐galactopyranosyl‐*(*1 → 4*)*‐β‐d‐glucopyranoside* (*3*′*‐FucLac‐tBoc **3**, and 6*′*‐FucLac‐tBoc **4**, and 6*′*‐FucLac‐tBoc **5***).

*p*NP‐α‐l‐Fuc (**1**; 51.3 mg; 180 μmol), Lac‐*t*Boc (**2**; 195 mg; 370 μmol; synthesized previously (Bojarová et al. 2019)), and α‐l‐f1*Pth*‐wt (0.53 mU mL^−1^; 0.31 mg) were incubated in 50 mM sodium‐phosphate buffer pH 8.0 at 37°C and 850 rpm (with the total reaction volume of 6 mL). In case of α‐l‐f1*Pth*‐S237V, the reaction mixture, with the total volume of 10 mL, contained *p*NP‐α‐l‐Fuc (**1**; 84 mg; 295 μmol), Lac‐*t*Boc (**2**; 195 mg; 605 μmol), and the mutant variant (0.53 mU mL^−1^; 5.1 mg). The progress of the reactions was monitored by TLC (*i*PrOH:water:NH_4_OH *aq*., 7:2:1) and by HPLC (TSKgel Amide‐80 column with PDA detector). The reactions were carried out for 33 h with α‐l‐f1*Pth*‐wt, or 168 h with α‐l‐f1*Pth*‐S237V, and were stopped by boiling for 5 min. The precipitated enzyme was removed by centrifugation (12,200 *g*, 10 min) and the supernatant was separated on a gel permeation chromatography column (26 × 1000 mm) loaded with the stationary phase of Biogel P‐2 (Bio‐Rad, UK) using water as a mobile phase at an elution rate of 7.2 mL h^−1^ at ambient temperature. The collected fractions were analysed by TLC, and the product‐containing fractions were pooled and lyophilized.

In the reaction catalysed by α‐l‐f1*Pth*‐wt, three products originated that were further separated to homogeneity by semi‐preparative HPLC on TSKgel Amide‐80 column (300 × 7.8 mm, 5 μm) preceded by a TSKgel Amide‐80 Guardgel (3.2 × 15 mm; Tosoh, Japan), using isocratic elution in 77% (*v/v*) acetonitrile in water as a mobile phase at a flow rate of 2.0 mL min^−1^, at 25°C, and detection at 200 nm. The structure of the three obtained products was confirmed by NMR: 3′‐FucLac‐*t*Boc (**3**; 6.5 mg; 9.4 μmol; 3.2% yield), 6′‐FucLac‐*t*Boc (**4**; 5.3 mg; 7.7 μmol; 2.6% yield), and 6‐FucLac‐*t*Boc (**5**; 5.4 mg; 7.8 μmol; 2.7% yield).

The reaction catalysed by α‐l‐f1*Pth*‐S237V provided a single product 3′‐FucLac‐*t*Boc (**3**; 30.6 mg; 15 μmol; 8.3% isolated yield). The analytical data for all three products can be found in the Supporting Information (Tables S2–S4, Figures S5–S7).

*Preparative Transglycosylation Reaction: α‐l‐fucopyranosyl‐*(*1 → 3*′*/6*′)*‐β‐d‐galactopyranosyl‐*(*1 → 4*)*‐D‐glucopyranose* (*3*′*‐FucLac **7a**, and 6*′*‐FucLac **7b***).

*p*NP‐α‐l‐Fuc (**1**; 51.3 mg; 180 μmol), lactose (**6**; 123.2 mg; 685 μmol), and α‐l‐f1*Pth*‐S237V (1.06 mU mL^−1^; 10.2 mg) were incubated in 50 mM sodium‐phosphate buffer pH 8.0. The reaction was carried out at 37°C and 850 rpm for 72 h in a total reaction volume of 6.0 mL. The progress of the reaction was monitored by TLC (propane‐2‐ol:water:NH_4_OH *aq*., 7:2:1, *v/v/v*) and HPLC (Luna NH_2_ column) with ELSD detector. The reaction was stopped by boiling for 5 min and centrifuged (12,200 *g*, 10 min) to remove the precipitated enzyme. The supernatant was then loaded onto gel permeation chromatography column (26 × 1000 mm) loaded with the stationary phase Biogel P‐2 using water as a mobile phase at an elution rate of 7.4 mL h^−1^ at ambient temperature. The fractions were collected, analysed by TLC, and the fractions containing the products were combined and lyophilized. The structure of the obtained products was confirmed by NMR to detect a mixture of two products 3′‐FucLac **7a** and 6′‐FucLac **7b** in a molar ratio of 72:28 (13.4 mg; 27 μmol in total; 15.3% isolated yield). For the structural analysis, see the Supporting Information (Tables S5 and S6, Figure S8).

### 2.9 NMR Analysis of Synthesized Compounds **3**, **4**, **5**, and **7**

NMR spectra of compounds **3**, **4**, **5**, and **7** (as the mixture of both **7a** and **7b** anomers) were acquired on a BrukerAvance III 700 MHz spectrometer (Bruker BioSpin, Germany) in D_2_O (99.96 atom% D, VWR Chemicals, Belgium) at 30°C. Residual signal of D_2_O (*δ*_H_ 4.732 ppm) was used for the proton spectra reference; carbon chemical shifts were referenced to the signal of acetone (*δ*_C_ 30.50 ppm). The individual monosaccharide units were assigned using COSY, HSQC, and 1D‐TOCSY experiments performed using manufacturer's software; their anomeric configuration was deduced from the magnitude of *J* (H1,H2) coupling constant. Glycosidic linkage was confirmed by the HMBC correlation between the involved carbon and the adjacent anomeric proton.

## 3 Results and Discussion

In our previous studies, we reported two recombinant isoenzymes of α‐l‐fucosidase from *Paenibacillus thiaminolyticus* (Benešová et al. 2015, 2013) capable of transferring an α‐l‐fucosyl moiety to a wide variety of acceptor molecules. Although the yields of the transglycosylation reactions were not high in all cases, these isoenzymes demonstrated significant potential in the synthesis of bioactive fucosylated compounds, such as fucosylated human milk oligosaccharides (HMOs).

Building on these promising findings, we tried to improve the transglycosylation abilities of α‐l‐fucosidase from *P. thiaminolyticus* isoform 1 (α‐l‐f1*Pth*‐wt) (Benešová et al. 2013) using site‐directed mutagenesis to achieve an even higher transglycosylation efficiency. Following these modifications, we analysed in detail the regioselectivity and transglycosylation capability of α‐l‐f1*Pth*‐wt and its mutated variants in the preparative production of fucosylated lactose and its functionalized counterparts.

### 3.1 Design and Preparation of α‐l‐f1 *Pth*‐wt Mutants

To enhance the transglycosylation abilities of α‐l‐f1*Pth*‐wt, we designed several mutant variants of this enzyme. Initially, we discovered that α‐l‐f1*Pth*‐wt shares 29% identity with α‐l‐fucosidase from *Thermotoga maritima* (*Tm*αFuc), from which α‐l‐transfucosidase was successfully prepared (Osanjo et al. 2007). Despite the relatively low overall identity, the catalytic domains of both enzymes appeared to be well conserved, as revealed by sequence alignment performed using Clustal Omega (Supporting Information, Figure S1). Moreover, both enzymes occur in a hexameric form. By comparing the three‐dimensional structures of *Tm*αFuc (PDB ID: 1HL8) (Sulzenbacher et al. 2004) and α‐l‐f1*Pth*‐wt (PDB ID: 6GN6) (Kovalova et al. 2019), we identified amino acid positions in α‐l‐f1*Pth*‐wt corresponding to the three mutations in *Tm*αFuc (T264A, Y267F, and L322P) located in the second amino acid shell of the active site, which were confirmed to directly increase the transglycosylation/hydrolysis ratio of *Tm*αFuc.

Although these amino acids do not directly interact with the substrate, they can significantly influence enzyme activity by modulating the positions of first‐shell residues, thereby affecting substrate binding or selectivity, product release, enzyme stability, and other biochemical characteristics.

**FIGURE 1:**
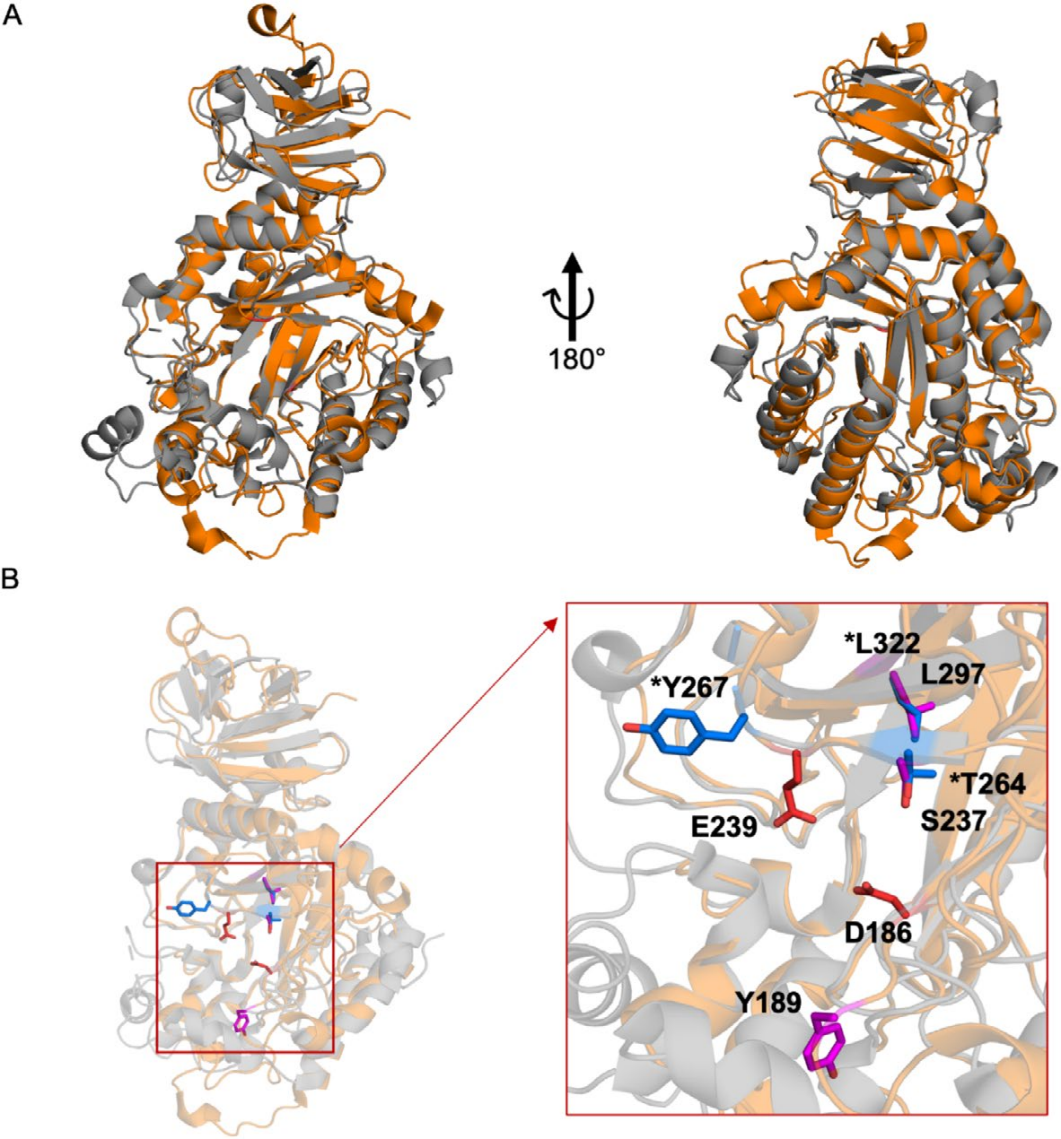
Comparison of the structures of α‐l‐f1*Pth*‐wt and *Tm*αFuc. (A) Superposition of the structures of a single chain of α‐l‐f1*Pth*‐wt (orange) and *Tm*αFuc (grey). (B) Position of the active site within the single chain of the compared structures, with catalytic residues of α‐l‐f1*Pth*‐wt coloured in red and the positions of individual residues selected for mutations illustrated as sticks (residues of α‐l‐f1*Pth*‐wt in magenta and of *Tm*αFuc in blue marked with an asterisk).

The superposition of both structures identified residues S237 and L297 in α‐l‐f1*Pth*‐wt corresponding to the mutations found in the transglycosylating mutants of *Tm*αFuc. The residue Y267 of *Tm*αFuc is located within a partially disordered loop absent in the α‐l‐f1*Pth*‐wt structure. Instead, a different tyrosine residue, Y189, was selected for mutagenesis, positioned in the second amino acid shell on the opposite side of the active site cavity (Figure 1). Although Y189 is located on the other side of the active site cavity, it can influence the entry of the acceptor into the active site, similarly to Y267 in *Tm*αFuc. With regard to the presence of the same functional group in the side chain, its position in the second amino acid shell of the active site, and its comparable distance from the catalytic center (though to a different active site residue), this residue represents an attractive target for a possible modulation of transglycosylation activity.

Despite the structural similarity between α‐l‐f1*Pth*‐wt and *Tm*αFuc, it is important to highlight a key difference that may significantly affect the alteration of the transglycosylation/hydrolysis activity ratio as well as the enzyme kinetics. In the tertiary structure of the C‐terminal domain of α‐l‐f1*Pth*‐wt, there is a 10‐residue loop containing a tryptophan residue at position 392. Trp392 complements the active site of the adjacent monomer within the native hexameric assembly (Kovalova et al. 2019). Together with Trp35, it forms a carbohydrate‐binding motif known as the tryptophan box. This distinctive structural feature suggests that the architecture of the active site in α‐ l‐f1*Pth*‐wt is more complex than that of *Tm*αFuc.

First, the designed mutations were inserted into the gene of α‐l‐f1*Pth*‐wt in the expression plasmid pET16b‐α‐l‐f1*Pth*‐wt using specific primers for PCR. The recombinant α‐l‐f1*Pth*‐wt and its mutant enzymes, all in fusion with a polyhistidine tag, were produced in *E. coli* BL21 (DE3) and purified by affinity chromatography on Ni‐NTA agarose. In all cases, the total protein yield was approximately 10 mg obtained from culture grown in 1 L of LBA medium. The L297P mutant was directed to inclusion bodies, even though we optimized the production and cell disruption conditions (as described in Methods). A possible explanation is that the L297P mutation, which introduces the cyclic amino acid proline, disrupts the local β‐sheet structure (Kovalova et al. 2019), thus impairing proper protein folding and leading to its accumulation in inclusion bodies.

### 3.2 Characterization of the Hydrolytic and Transglycosylation Capabilities of Mutant Enzymes

The hydrolytic activities of purified enzymes (α‐l‐f1*Pth*‐wt and its five successfully prepared mutant variants) were determined using the chromogenic substrate *p‐*nitrophenyl α‐l‐fucopyranoside (*p*NP‐α‐l‐Fuc). All variants displayed substantially decreased hydrolytic activity compared to the wild‐type enzyme (Table 1).

**TABLE 1:** Hydrolytic activities of α‐l‐f1*Pth*‐wt and its mutant variants.

Hydrolytic activity	α‐l‐f1*Pth*‐wt	S237A	S237G	S237P	S237V	Y189F
[U mg^−1^]	443 ± 18	301 ± 16	253 ± 11	14 ± 1	35 ± 3	79 ± 3

*Note:* Data are shown as average values with their standard deviations calculated from three repetitions of the measurement.

The hydrolytic activities of the resulting variants showed a wide range of reductions compared to α‐l‐f1*Pth*‐wt (443 ± 18 U mg^−1^), indicating distinct structural and functional consequences of each substitution. Regarding the position S237, the first mutant S237A retained moderate hydrolytic activity (301 ± 16 U mg^−1^), indicating that the loss of the hydroxyl group had only a limited effect on the water accessibility to the catalytic center. The S237G variant showed a greater decline (253 ± 11 U mg^−1^), probably due to increased backbone flexibility introduced by glycine, which is the smallest amino acid residue. In contrast, the S237P mutant exhibited a severe loss of activity (14 ± 1 U mg^−1^), consistent with the known structural rigidity of proline, which can dramatically destabilize local secondary structures. Similarly, the S237V variant (35 ± 3 U mg^−1^) introduced a bulkier hydrophobic side chain, which could cause steric hindrance or alter substrate positioning, leading to reduced catalytic efficiency. Interestingly, the Y189F mutation also resulted in a significant reduction in hydrolytic activity (79 ± 3 U mg^−1^). This suggests that the hydroxyl group of tyrosine at this position may play a role in modulating the microenvironment of the active site, possibly by influencing water accessibility. The most pronounced reductions in hydrolytic activity were observed in the S237P, S237V, and Y189F variants, which were therefore selected for further experiments.

To evaluate the impact of inserted mutations on the transglycosylation/hydrolysis activity ratio, transglycosylation abilities of the wild‐type enzyme and the selected mutant variants (S237P, S237V, Y189F) were compared. 50 mM *p*NP‐α‐l‐Fuc was used as the donor of l‐fucosyl moiety. We used 300 mM lactose as the acceptor molecule, as it is a well‐established precursor in the biosynthesis of various HMOs (McDonald et al. 2022).

The quantitative determinations of transglycosylation products were carried out by HPLC using Supelcogel Ca column suitable for oligosaccharide separation coupled with UV/VIS and ELSD detectors. To determine the optimal reaction time for achieving the maximum yields of fucosylated lactose, the reaction rates were monitored at various time intervals. The highest concentrations of the overall transglycosylation products were then quantified using the internal standard method (Table 2).

**TABLE 2:** Maximum overall yields of fucosylated lactose (a mixture of all regioisomers) and transglycosylation/hydrolysis ratios calculated at the given reaction times for the wild‐type enzyme α‐l‐f1*Pth*‐wt and the selected mutant variants.

Enzyme variant	Maximum yield [mmol L^−1^]	Reaction time at maximum yield	Transglycosylation/hydrolysis ratio^a^
α‐l‐f1*Pth*‐wt	9.5	5 min	0.02
S237P	9.5	6 h	0.68
S237V	14.3	1 h	0.41
Y189F	6.9	2 h	0.09

^a^ The transglycosylation/hydrolysis ratios were calculated by dividing the maximum product yield (concentration of fucosylated lactose at the given reaction time) by the average hydrolytic activities of the respective enzyme (Table 1).

The wild‐type enzyme α‐l‐f1*Pth*‐wt exhibited extensive and rapid hydrolysis, achieving its maximum transglycosylation yield in just 5 min, significantly faster than any of the mutant variants. However, its transglycosylation/hydrolysis activity ratio was notably low (0.02), indicating that hydrolysis was the predominant reaction. In contrast, all three mutant variants demonstrated a clear shift in their catalytic preference toward transglycosylation. The S237P variant matched the transglycosylation yield of the wild‐type enzyme (9.5 mmol L^−1^) but required a considerably longer reaction time (6 h). Although the shift in the transglycosylation/hydrolysis ratio was the most pronounced among all variants (0.68), corresponding to a 34‐fold increase, no improvement in the overall product yield was observed. The S237V mutant showed the highest overall fucosylated lactose yield (14.3 mmol L^−1^) and a favourable transglycosylation/hydrolysis ratio of 0.41, representing more than a 20‐fold increase. These results indicate that the introduction of a bulkier hydrophobic side chain may facilitate the acceptor molecule entry at the expense of water. By contrast, the Y189F variant exhibited a modest yield (6.9 mmol L^−1^) and a relatively low transglycosylation/hydrolysis ratio (0.09), despite its significantly reduced hydrolytic activity. This suggests that while the mutation affects the catalytic environment, it may not sufficiently favour the transglycosylation pathway, possibly due to suboptimal acceptor positioning or limited stabilization of the glycosyl‐enzyme intermediate. Given its highest overall transglycosylation yield, the S237V mutant (α‐l‐f1*Pth*‐S237V) was chosen for subsequent experimental investigation.

**FIGURE 2:**
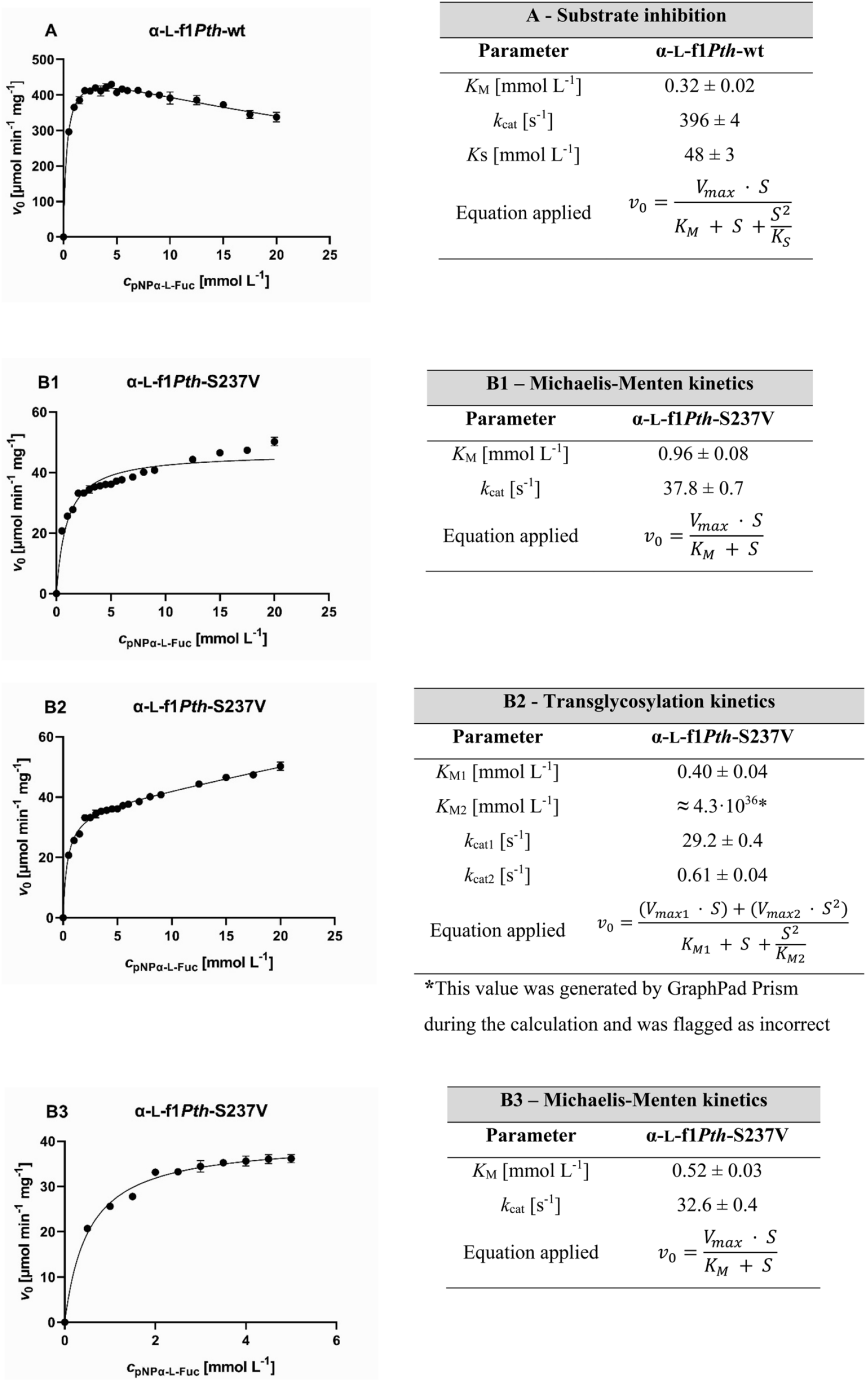
Dependence of the initial reaction rate (*v*_0_) on the concentration of *p*NP‐α‐l‐Fuc (*c*_*p*NP‐α‐l‐Fuc_) and comparison of the calculated kinetic parameters of α‐l‐f1*Pth*‐wt and α‐l‐f1*Pth*‐S237V. (A) kinetics with substrate inhibition of α‐l‐f1*Pth*‐wt, (B1) Michaelis–Menten kinetics for α‐l‐f1*Pth*‐S237V, (B2) transglycosylation kinetics of α‐l‐f1*Pth*‐S237V, and (B3) Michaelis–Menten kinetics of α‐l‐f1*Pth*‐S237V within the substrate concentration range 0–5 mmol·L^−1^. Kinetic parameters: *K*_M_ is the Michaelis constant, *k*_cat_ is the turnover number, and *K*_*S*_ is the inhibition constant; for the transglycosylation kinetics, *k*_cat1_ is the turnover number and *K*_M1_ is the Michaelis constant for hydrolysis, whereas *k*_cat2_ together with *K*_M2_ are analogous parameters for transglycosylation.

Furthermore, we compared the hydrolytic reaction kinetic parameters for α‐l‐f1*Pth*‐wt and α‐l‐f1*Pth*‐S237V using the *p*NP‐α‐l‐Fuc substrate (Figure 2).

In our previous work (Benešová et al. 2013), we observed substrate inhibition at higher substrate concentrations for α‐l‐f1*Pth*‐wt. This observation was confirmed in the current study, where we also assume that the contribution of the transglycosylation reaction to the shape of the curve is negligible (Figure 2A).

**SCHEME 1:**
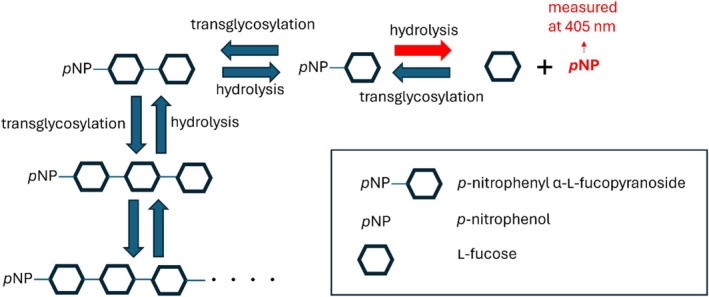
Schematic illustration of the dual catalytic activity of α‐l‐fucosidase, highlighting simultaneous hydrolysis and transglycosylation reactions. Both processes occur concurrently, sharing available substrates and intermediates, leading to distinct products through competing pathways. The enzymatic hydrolytic activity of *p*NP‐α‐l‐Fuc is monitored by increasing absorbance at 405 nm, corresponding to the released *p*‐nitrophenol (highlighted in red).

By contrast, a different behaviour was observed for α‐l‐f1*Pth*‐S237V (Figure 2B). As expected, this variant exhibited significantly lower maximum hydrolysis rates at substrate concentrations comparable to those of the wild‐type enzyme. Although the data may initially appear to fit the curve described by the Michaelis–Menten equation, this impression is misleading, as demonstrated by the discrepancy observed when the data points are plotted against the corresponding theoretical curve (Figure 2B1). It is important to consider that the shape of the resulting curve may be influenced by three concurrent processes: hydrolysis, substrate inhibition (if still present after the mutation), and transglycosylation. However, the chosen method only allows for detection of *p*‐nitrophenol release. Additionally, the situation is further complicated by the fact that the transglycosylation products can act as substrates for hydrolysis or as acceptors in subsequent transglycosylation steps. Nevertheless, the enzyme affinity and kinetic constants for these alternative substrates may differ considerably. Moreover, the rate of the transglycosylation reaction (and thus the transglycosylation/hydrolysis activity ratio) varies depending on the substrate concentration present in the reaction mixture. The complexity of this process is illustrated in Scheme 1.

As discussed above, fitting the obtained data to the simple Michaelis–Menten model (Figure 2B1) proved to be inaccurate (*K*_M_ = 0.96 ± 0.08 mmol L^−1^, *k*_cat_ = 37.8 ± 0.7 s^−1^). To better capture the complexity of the system, we applied the equation for simultaneous hydrolysis and transglycosylation (Figure 2B2) as previously employed by Nguyen et al. (2014) in reference to the findings of Gusakov et al. (1984) and Kawai et al. (2004). This alternative approach provided a noticeably improved fit (*K*_M1_ = 0.40 ± 0.04 mmol L^−1^, *k*_cat1_ = 29.2 ± 0.4 s^−1^, *K*_M2_ = 4.3 × 10^36^ mmol L^−1^ and *k*_cat2_ = 0.61 ± 0.04 s^−1^), with the resulting curve closely matching the experimental data. However, it yielded physically unrealistic parameters for the transglycosylation reaction (an extremely high Michaelis constant *K*_M2_ together with an implausibly low turnover number *k*_cat2_). This indicates that the model does not adequately reflect the actual enzymatic mechanism. The discrepancy likely reflects mechanistic features not captured by the model, such as substrate inhibition or multi‐step formation of transglycosylation products. A more rigorous kinetic treatment would require direct quantification of transglycosylation products during the reaction time course, which was beyond the scope of this study. In contrast, the *K*_M1_ value for hydrolysis (0.40 ± 0.04 mmol L^−1^) appears to correspond well to the kinetic behaviour of our enzyme. As a result, we consequently used this model only to illustrate the qualitative shift in catalytic preference between hydrolysis and transglycosylation, rather than as a source of reliable absolute kinetic constants.

Given that transglycosylation reactions and substrate inhibition are expected to be minimal at low substrate concentrations, we evaluated the hydrolysis kinetics using the Michaelis–Menten analysis within the 0–5 mmol L^−1^ substrate concentration range (Figure 2B3) to obtain reliable estimates of the kinetic parameters. Fitting the data to the classical Michaelis–Menten model supported our hypothesis that competing reactions exert minimal influence at low substrate concentrations and the following kinetic parameters were obtained: *K*_M_ = 0.52 ± 0.03 mmol L^−1^ and *k*_cat_ = 32.6 ± 0.4 s^−1^.

Based on all obtained Michaelis constant values, it can be inferred that α‐l‐f1*Pth*‐S237V exhibits only a slightly reduced affinity for the *p*NP‐α‐l‐Fuc substrate. This finding is also in agreement with the results of Osanjo et al. (2007) (Osanjo et al. 2007), who reported a slight increase in the *K*_M_ value at 40°C for the same substrate—from 0.020 to 0.065 mmol L^−1^—in the corresponding T264A mutant of *Tm*αFuc. Theoretically, it is possible to speculate that the presence of valine may contribute to the increased *K*_M_ value. Since this amino acid is more hydrophobic than serine and lacks a hydroxyl group, it may hinder substrate access to the active site, thereby reducing the apparent enzyme affinity.

For a more precise description of enzyme kinetics, it would be necessary to analyse the concentration of all components of the reaction mixture as a function of substrate concentration. However, the aim of this work was not to conduct a detailed kinetic study of the characteristics of the mutated enzyme.

### 3.3 Transglycosylation Capability of α‐l‐f1*Pth* ‐Wt and the S237V Variant

**SCHEME 2:**
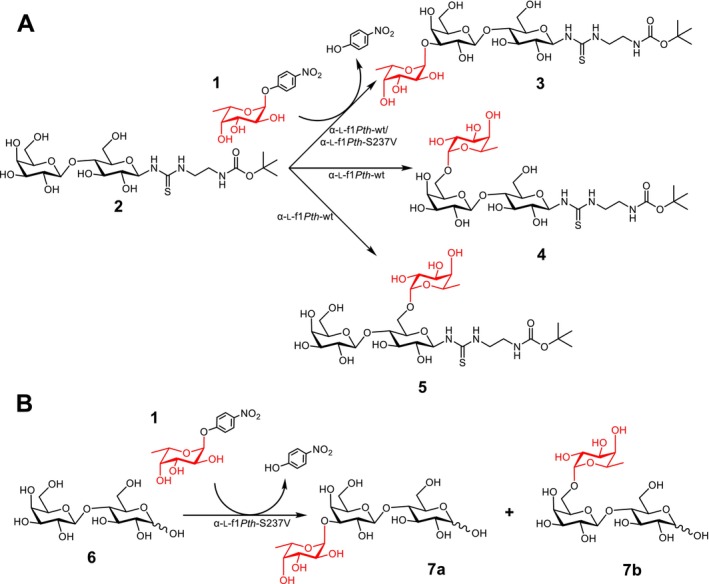
Enzymatic synthesis of (**A**) functionalized trisaccharides 3′‐FucLac‐*t*Boc (**3**; Fucα3Galβ4Glc‐*t*Boc), 6′‐FucLac‐*t*Boc (**4**; Fucα6Galβ4Glc‐*t*Boc), 6‐FucLac‐*t*Boc (**5**; Galβ4(Fucα6)Glc‐*t*Boc) and (**B**) trisaccharides 3′‐FucLac (**7a**; Fucα3Galβ4Glc) and 6′‐FucLac (**7b**; Fucα6Galβ4Glc) catalysed by α‐l‐f1*Pth*‐wt and/or α‐l‐f1*Pth*‐S237V using *p*NP‐α‐l‐Fuc (**1**) as a donor and lactosyl‐*t*Boc (**2**) or lactose (**6**) as an acceptor.

The wild‐type enzyme α‐l‐f1*Pth*‐wt and the most promising mutant variant α‐l‐f1*Pth*‐S237V were tested for their potential to synthesize free and C‐1 functionalized fucosyllactose as a substantial part of the HMOs cohort (Scheme 2). Lactose (**6**) and the C‐1 functionalized substrate (*tert*‐butoxycarbonylamido)ethylthioureidyl β‐D‐galactopyranosyl‐(1→ 4)‐β‐D‐glucopyranoside (Lac‐*t*Boc or lactosyl‐*t*Boc; **2**) were used as acceptors to investigate enzyme regioselectivity and its ability to tolerate C‐1 modification in the acceptor molecule. Lactosyl‐*t*Boc was synthesized based on the previously described synthetic procedure for GlcNAc‐*t*Boc (Bojarová et al. 2019) with an overall yield of 23% and was structurally characterized (Slámová et al. 2023). The presence of the *t*Boc linker at C‐1 enables straightforward immobilization of the trisaccharide to biosensor surfaces or conjugation to macromolecules, creating multivalent presentation (Bojarová et al. 2018).

To assess suitable conditions for preparative transfucosylation reactions, the donor/acceptor ratio was investigated on an analytical scale using 30 mM *p*NP‐α‐l‐Fuc as a donor, 60–300 mM acceptor, and α‐l‐f1*Pth*‐wt. The analysis showed the best performance at an acceptor concentration of 60 mM with a donor/acceptor ratio of 1:2, making α‐l‐f1*Pth*‐wt a more convenient tool for synthetic application than *Tm*αFuc, which required the donor/acceptor ratio of approximately 1:1000 (Guzmán‐Rodríguez et al. 2018a). Lactose was successfully fucosylated by the action of α‐l‐f1*Pth*‐wt. However, due to the very low yield of the desired products, we were unable to isolate them in sufficient quantity and purity for structural characterization by NMR (Table 3). In contrast, the reaction catalysed by α‐l‐f1*Pth*‐S237V resulted in a significantly increased product formation, as confirmed by HPLC analysis, and allowed the isolation of a mixture of 3′‐and 6′‐fucosyllactose with an overall yield of 15.3%.

**TABLE 3:** Isolated yields of fucosylated products catalysed by α‐l‐f1*Pth* and α‐l‐f1*Pth*‐S237V using the *p*NP‐α‐l‐Fuc donor and lactose or Lac‐*t*Boc acceptor.

Enzyme	Donor	Acceptor	Product	Isolated yield of regioisomers^a^	Overall isolated yield^a^
α‐l‐f1*Pth*‐wt	*p*NP‐α‐l‐Fuc (**1**; 51 mg)	Lac (**6**; 123 mg)	traces of products	n.d.^b^	n.d.^b^
α‐l‐f1*Pth*‐wt	*p*NP‐α‐l‐Fuc (**1**; 84 mg)	Lac‐*t*Boc (**2**; 319 mg)	3′‐FucLac‐*t*Boc (**3**)/6′‐FucLac‐*t*Boc (**4**)/6‐FucLac‐*t*Boc (**5**)	6.5 mg (3.2%)/5.3 mg (2.6%)/5.4 mg (2.7%)	17.2 mg (8.5%)
α‐l‐f1*Pth*‐S237V	*p*NP‐α‐l‐Fuc (**1**; 51 mg)	Lac (**6**; 123 mg)	3′‐FucLac (**7a**)/6′‐FucLac (**7b**)	13.4 mg (15.3%)^c^	13.4 mg (15.3%)
α‐l‐f1*Pth*‐S237V	*p*NP‐α‐l‐Fuc (**1**; 51 mg)	Lac‐*t*Boc (**2**; 195 mg)	3′‐FucLac‐*t*Boc (**3**)	10.3 mg (8.3%)	10.3 mg (8.3%)

^a^ The product yields were calculated using the following formula: yield [%] = (molar amount of isolated product)/(theoretical molar amount of product corresponding to the molar amount of donor used) × 100.

^b^ n.d., not determined.

^c^ Products **7a** and **7b** were isolated as a mixture and their molar ratio was found to be 72:28 by NMR.

The transglycosylation reaction with α‐l‐f1*Pth*‐wt using C‐1 functionalized lactosyl‐*t*Boc proceeded much more efficiently. A mixture of three products was isolated by gel chromatography and further purified by semi‐preparative HPLC to obtain pure FucLac‐*t*Boc regioisomers: 3′‐FucLac‐*t*Boc, 6′‐FucLac‐*t*Boc and 6‐FucLac‐*t*Boc, in an overall isolated yield of 8.5%. These results indicate that α‐l‐f1*Pth*‐wt exhibits relatively low regiospecificity during the transglycosylation reaction with the lactosyl‐*t*Boc acceptor.

In contrast, α‐l‐f1*Pth*‐S237V exclusively generated a single regioisomer, 3′‐FucLac‐*t*Boc, with an isolated yield of 8.3%. Despite producing only one product, the overall yield remained comparable to that of the wild type, as both enzymes displayed similar catalytic activities. This outcome highlights a substantial gain in regioselectivity conferred by the S237V substitution. The presence of valine likely alters the geometry of the active site in a way that restricts acceptor orientation, thereby favouring a single, well‐defined transglycosylation pathway.

Using site‐directed mutagenesis, the α‐l‐f1*Pth*‐S237V variant was successfully engineered to efficiently utilize lactose as an acceptor, resulting in substantial yields of 3′‐FucLac and 6′‐FucLac. Moreover, it exhibits altered regioselectivity by producing a single product (3′‐FucLac‐tBoc) when a functionalized lactose was used as an acceptor, in contrast to the wild‐type enzyme that generated a mixture of three regioisomers (3′‐FucLac‐*t*Boc, 6′‐FucLac‐*t*Boc, and 6‐FucLac‐*t*Boc). These findings are consistent with the promiscuity commonly observed in *α*‐L‐fucosidase‐mediated transglycosylation reactions and highlight the importance of engineering enzymes with enhanced regioselectivity for targeted glycosynthetic applications.

The enhanced regioselectivity observed for the α‐l‐f1*Pth*‐S237V variant aligns with broader trends reported for engineered GH29 α‐l‐fucosidases, where targeted mutations can markedly reshape the product profile of transglycosylation reactions. The structure guided mutagenesis of *Fusarium graminearum* α‐l‐fucosidase demonstrated that substitutions positioned near the acceptor binding region can redirect the enzyme activity toward the preferred regioisomer, even without markedly increasing its overall transglycosylation yields, a behaviour closely paralleling the regioselectivity shift observed for α‐l‐f1*Pth*‐S237V (Zeuner et al. 2020). Another example is α‐l‐fucosidase Fuc2358, isolated from the intestinal metagenome of breastfed infants, whose site‐directed mutagenesis yielded variants that shifted the reaction profile toward markedly higher 2′‐FL production relative to donor hydrolysis (Moya‐Gonzálvez et al. 2024). Similarly, the semi‐rational engineering of α‐l‐fucosidase from *Bacteroides fragilis* generated variants that shifted the reaction outcome from a mixture of fucosyl‐*N*‐acetylglucosamine regioisomers almost exclusively to the α1,3‐linked product, demonstrating that even single residue substitutions can precisely control how the acceptor is positioned in the catalytic pocket and thereby dictate the resulting glycosidic linkage (Liu et al. 2025). Comparative analyses across GH29 enzymes further highlight that regioselectivity is not a fixed property but varies widely among natural homologues, reflecting differences in active site topology and substrate binding dynamics that can be exploited through protein engineering (Perna et al. 2023).

Direct comparison of the yields obtained in this study with previously published data on HMO precursor synthesis using α‐l‐fucosidase systems is quite challenging. We therefore emphasize the importance of reporting not only conversion rates but also the final yields of purified target products. For example, *Tm*αFuc has been shown to produce 2′‐FL from *p*NP‐α‐l‐Fuc and lactose with apparent conversions of 25%–33% and up to 40% under optimized conditions (Guzmán‐Rodríguez et al. 2018a). However, these values reflect reaction conversions quantified by, e.g., chromatographic methods, rather than the actual recovery of the purified product. A similar reliance on conversion data was found in studies of metagenome‐derived GH29 *α*‐l‐fucosidases (Lezyk et al. 2016) and the *Lactobacillus rhamnosus GG* enzyme (Escamilla‐Lozano et al. 2019), where conversions of up to 35% were reported without corresponding isolated yields. An outstanding exception is the marine α‐l‐fucosidase OUC‐Jdch16, which produced 2′‐FL from *p*NP‐α‐l‐Fuc and lactose in up to 92% isolated yield (Zhou et al. 2022), demonstrating that a very high preparative efficiency is achievable under favourable conditions. The formation of multiple regioisomers reveals the limitation of reporting conversions alone, as they may include undesired products. Quantification of isolated yields offers a more accurate measure of synthetic performance and highlights the value of *α*‐l‐fucosidase engineering for improved regioselectivity.

The availability of efficient and economically sustainable tools for synthesizing defined fucosylated oligosaccharides is becoming increasingly important for the food industry, medicine, and basic research. Although HMOs are already used in infant formula, only a limited number of structures are currently included. In contrast, human breast milk contains a highly heterogeneous mixture of fucosylated and non‐fucosylated oligosaccharides, whose individual and synergistic biological effects remain poorly understood. Therefore, developing reliable methods for synthesizing a broad range of fucosylated structures is essential because of their potential biological relevance despite the low natural abundance, but also because of their broader roles in key physiological processes. Beyond their value in studying HMO function and infant health, fucosylated molecules, including glycoconjugates, are involved in numerous biological pathways that can only be explored in detail if their core structures are accessible through efficient synthesis.

The results of our research indicate that site‐directed mutagenesis is a promising approach for designing enzymes suitable for the synthesis of structurally defined oligosaccharides, such as in our case when a single amino acid substitution transformed an average catalyst into a selective synthetic tool.

## 4 Conclusions

Human milk oligosaccharides (HMOs) comprise a structurally diverse group of over 200 compounds that perform a range of biological functions, including promoting healthy infant development, supporting the growth of beneficial gut microbiota, modulating immune responses, and providing protection against pathogenic microorganisms (Yu et al. 2025). Despite decades of intensive research into HMOs, many questions remain unanswered. Although some HMOs have already been approved as food additives for use in infant formula, their synthesis is still complicated. Consequently, there is a continuous demand for innovative approaches that enable the regioselective, economically feasible, and scalable synthesis of HMOs. Moreover, the development of synthetic pathways for novel HMO structures with potential applications in the food industry, medicine, and basic research remains a major focus. Importantly, the biological activity of individual oligosaccharides within this heterogeneous mixture is not yet fully understood, and their specific effects on human physiology have not yet been comprehensively elucidated.

In this study, we demonstrate that semi‐rational engineering of α‐l‐fucosidase iso1 from *Paenibacillus thiaminolyticus* (α‐l‐f1*Pth*‐wt) enables its functional tuning toward transglycosylation over hydrolysis, facilitating the efficient synthesis of the structurally complex target molecule fucosyllactose and its functionalized derivatives. This advancement is highly relevant for both the food industry and basic research, since lactose, the core structure of HMOs, is modified through various combinations of five distinct monosaccharide units: glucose, galactose, *N*‐acetylneuraminic acid, *N*‐acetylglucosamine, and fucose.

The site‐directed mutagenesis of α‐l‐f1*Pth*‐wt led to the identification of the S237V variant with markedly enhanced transfucosylation activity and exclusive regioselectivity. This variant enabled the efficient synthesis of 3′‐and 6′‐fucosyllactose, as well as a 3′‐functionalized derivative bearing a (*tert*‐butoxycarbonylamido)ethylthioureidyl linker. To date, the synthesis of fucosylated HMOs or lactose derivatives using α‐l‐fucosidase has been limited to unmodified acceptors such as lactose; the use of chemically modified acceptors has not yet been reported. Whereas efficient fucosylation of unmodified lactose is directly applicable to the (scalable) production of HMOs for infant formulas and functional foods, including prebiotics, the structurally defined functionalized fucosylated glycans, obtained through this biocatalytic approach, hold promise for applications in glycoengineering, biosensor development, or HMOs‐based diagnostics. This includes, for example, the development of fucosylated glycan arrays for specific recognition by lectins, microorganisms, or enzymes of biomedical interest. Functionalization of fucosylated glycans readily enables multivalent presentation, which is valuable for constructing glycomaterials that can serve as anti‐infective and immunomodulatory agents or anti‐adhesion therapeutics designed to decoy pathogen receptors binding fucosylated host glycans.

Our findings underscore the effectiveness of structure‐guided mutagenesis in modulating glycosidase activity, thereby expanding the biocatalytic toolbox for the synthesis of HMO analogues and other bioactive fucosylated glycans. This work highlights the potential of site‐directed mutagenesis as a powerful strategy for developing tailor‐made enzymatic tools for regioselective glycoside synthesis and advances the state of the art in glycoengineering and applied biocatalysis.

## Author Contributions

**Patricie Vodičková:** methodology, investigation, data curation, writing – original draft, writing – review and editing. **Lucie Klimešová:** methodology, investigation, data curation. **Pavlína Nekvasilová:** methodology, investigation, data curation, writing – original draft. **Lucie Petrásková:** methodology, investigation, data curation. **Helena Pelantová:** methodology, investigation, data curation. **Terézia Kovaľová:** investigation, data curation. **Petra Lipovová:** conceptualization, resources, supervision, writing – review and editing. **Pavla Bojarová:** conceptualization, resources, funding acquisition, project administration, supervision, writing – original draft, writing – review and editing. **Eva Benešová:** conceptualization, methodology, investigation, data curation, resources, project administration, supervision, writing – original draft, writing – review and editing. All authors approve of the current version of the manuscript.

## Funding

This work was supported by Ministerstvo Školství, Mládeže a Tělovýchovy, LUC24024. Grantová Agentura České Republiky, 25‐16301K.

## Conflicts of Interest

The authors declare no conflicts of interest.

## Supporting information


**Appendix S1:** mbt270354‐sup‐0001‐Supinfo.docx.
**Figure S1:** Alignment of amino acid **s**equences of α‐l‐f1*Pth*‐wt and *Tm*αFuc.
**Figure S2:** Nucleotide sequence of the gene encoding α‐l‐fucosidase iso1 from *Paenibacillus thiaminolyticus* .
**Figure S3:** Gel after SDS‐PAGE of purified α‐l‐f1*Pth*‐wt and its mutated variants produced in *E. coli* BL21 (DE3) cells.
**Figure S4:** (A) Dependence of α‐L‐f1*Pth*‐wt activity on the concentration of DMF in the reaction and (B) stability of α‐l‐f1*Pth*‐wt after incubation with 10% DMF (*v/v*).
**Figure S5A:** mbt270354‐sup‐0001‐Supinfo.docx. ^1^H NMR spectrum of compound **3**.
**Figure S5B:** ^13^C NMR spectrum of compound **3**.
**Figure S5C:** HPLC chromatogram of compound **3**.
**Figure S5D:** MS spectrum (ESI^+^) of compound **3**.
**Figure S6A:** ^1^H NMR spectrum of compound **4**.
**Figure S6B:** ^13^C NMR spectrum of compound **4**.
**Figure S6C:** HPLC chromatogram of compound **4**.
**Figure S6D:** MS spectrum (ESI^+^) of compound **4**.
**Figure S7A:** ^1^H NMR spectrum of compound **5**.
**Figure S7B:** ^13^C NMR spectrum of compound **5**.
**Figure S7C:** HPLC chromatogram of compound **5**.
**Figure S7D:** MS spectrum (ESI^+^) of compound **5**.
**Figure S8A:** ^1^H NMR spectrum a mixture of compounds **7a** and **7b**.
**Figure S8B:** ^13^C NMR spectrum of a mixture of compounds **7a** and **7b**.
**Figure S8C:** HPLC chromatogram of a mixture of compounds **7a** and **7b**.
**Figure S8D:** MS spectrum (ESI^+^) of a mixture of compounds **7a** and **7b**.
**Table S1:** Nucleotide sequences of primers used for the preparation of plasmids containing genes encoding for potential α‐l‐transfucosidases.
**Table S2:** ^1^H and ^13^C NMR data of compound **3**.
**Table S3:** ^1^H and ^13^C NMR data of compound **4**.
**Table S4:** ^1^H and ^13^C NMR data of compound **5**.
**Table S5:** ^1^H and ^13^C NMR data of compound **7a**.
**Table S6:** ^1^H and ^13^C NMR data of compound **7b**.

## Data Availability

The data that supports the findings of this study are available in the Supporting Information of this article.
