# Supplementary material for: Transglycosylation Capabilities of Wild‐Type α‐l‐Fucosidase iso1 from Paenibacillus thiaminolyticus and Its Engineered Mutants: Preparation of Fucosylated Oligosaccharides
Source: Microb Biotechnol. 2026 Apr 23;19(4):e70354. doi: 10.1111/1751-7915.70354 (PMC13103868; doi:10.1111/1751-7915.70354)
Supplement: Supplementary file 1 — Appendix S1: mbt270354‐sup‐0001‐Supinfo.docx. Figure S1: Alignment of amino acid sequences of α‐l‐f1Pth‐wt and TmαFuc. Figure S2: Nucleotide sequence of the gene encoding α‐l‐fucosidase iso1 from Paenibacillus thiaminolyticus . Figure S3: Gel after SDS‐PAGE of purified α‐l‐f1Pth‐wt and its mutated variants produced in E. coli BL21 (DE3) cells. Figure S4: (A) Dependence of α‐L‐f1Pth‐wt activity on the concentration of DMF in the reaction and (B) stability of α‐l‐f1Pth‐wt after incubation with 10% DMF (v/v). Figure S5A: mbt270354‐sup‐0001‐Supinfo.docx. 1H NMR spectrum of compound 3. Figure S5B: 13C NMR spectrum of compound 3. Figure S5C: HPLC chromatogram of compound 3. Figure S5D: MS spectrum (ESI+) of compound 3. Figure S6A: 1H NMR spectrum of compound 4. Figure S6B: 13C NMR spectrum of compound 4. Figure S6C: HPLC chromatogram of compound 4. Figure S6D: MS spectrum (ESI+) of compound 4. Figure S7A: 1H NMR spectrum of compound 5. Figure S7B: 13C NMR spectrum of compound 5. Figure S7C: HPLC chromatogram of compound 5. Figure S7D: MS spectrum (ESI+) of compound 5. Figure S8A: 1H NMR spectrum a mixture of compounds 7a and 7b. Figure S8B: 13C NMR spectrum of a mixture of compounds 7a and 7b. Figure S8C: HPLC chromatogram of a mixture of compounds 7a and 7b. Figure S8D: MS spectrum (ESI+) of a mixture of compounds 7a and 7b. Table S1: Nucleotide sequences of primers used for the preparation of plasmids containing genes encoding for potential α‐l‐transfucosidases. Table S2: 1H and 13C NMR data of compound 3. Table S3: 1H and 13C NMR data of compound 4. Table S4: 1H and 13C NMR data of compound 5. Table S5: 1H and 13C NMR data of compound 7a. Table S6: 1H and 13C NMR data of compound 7b. [file MBT2-19-e70354-s001.docx]

**Supplementary Information**

**Transglycosylation Capabilities of Wild-Type α-l-Fucosidase Iso1 from *Paenibacillus thiaminolyticus* and Its Engineered Mutants: Preparation of Fucosylated Oligosaccharides**

Patricie Vodičková^a^, Lucie Klimešová^a,b^ , Pavlína Nekvasilová^b,c^, Lucie Petrásková^b^, Helena Pelantová^b^, Terézia Kovaľová^a^, Petra Lipovová^a^, Pavla Bojarová^b^, Eva Benešová^a,^*

^a^ Department of Biochemistry and Microbiology, University of Chemistry and Technology, Technická 5, 166 28 Prague 6, Czech Republic

^b^ Institute of Microbiology of the Czech Academy of Sciences, Vídeňská 1083, 142 00 Prague 4, Czech Republic

^c^ Department of Genetics and Microbiology, Faculty of Science, Charles University, Viničná 5, 128 00 Prague 2, Czech Republic

***Corresponding author**: Eva Benešová, Technická 5, 166 28 Prague 6, Czech Republic

[eva.benesova@vscht.cz](mailto:eva.benesova@vscht.cz), +420220445171

**Content:**

1. **Preparation and characterization of the α-l-f1*Pth*-wt mutant variants**
2. **Structural characterization of prepared transfucosylation products**
3. **Preparation and characterization of the α-l-f1*Pth*-wt mutant variants**


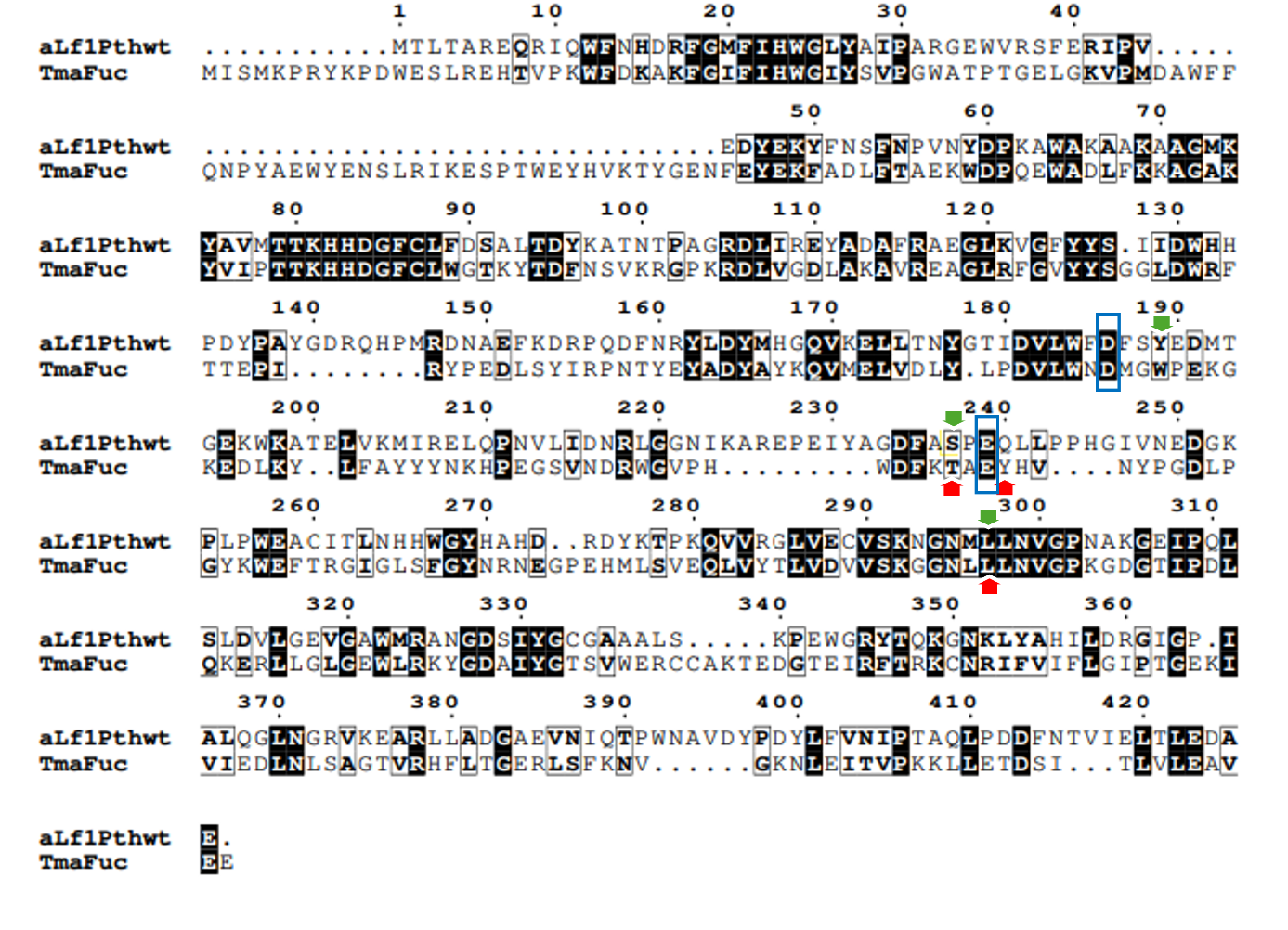


**Figure S1.** Alignment of amino acid **s**equences of α-l-f1*Pth*-wt and *Tm*αFuc. Amino acid residues modified in α-l-f1*Pth*-wt are marked with green arrows, amino acid residues of *Tm*αFuc, which served as the basis for the mutant design in this work, are marked with red arrows. Catalytic residues are highlighted with blue rectangles. Sequences were aligned using Clustal Omega (Sievers et al., 2011), and the illustration was generated using ESPript 3 (Robert and Gouet, 2014), followed by manual editing in Microsoft PowerPoint (https://www.microsoft.com/en-us/microsoft-365/powerpoint).

Sievers F., Wilm A., Dineen D., Gibson T. J., Karplus K., Li W., Lopez R., McWilliam H., Remmert M., Söding J., Thompson J. D., Higgins D. G.: Fast, scalable generation of high-quality protein multiple sequence alignments using Clustal Omega. Molecular Systems Biology, Volume 7, 2011, doi: 10.1038/msb.2011.75.

Robert X., Gouet P.: Deciphering key features in protein structures with the new ENDscript server. Nucleic Acids Research, Volume 42, 2014, doi: 10.1093/nar/gku316.

atgacgttaaccgcacgcgagcagcgtatacaatggttcaatcacgatcgcttcggcatgtttatccattggggattatatgcgattcccgcccggggcgaatgggttcgcagcttcgagcgcatcccggtcgaggattatgagaaatatttcaacagcttcaatccggtgaattatgatccgaaggcatgggccaaggccgccaaagccgcaggcatgaaatatgcggtcatgacgaccaagcaccatgacggcttctgtctgttcgacagcgccttgactgactataaagcgacgaatacgccggcgggccgcgatctgattcgcgaatatgcagacgctttccgggccgaaggactcaaggtcggcttctattactcgattatcgactggcatcatccggactatccggcctatggcgatcgccagcatccgatgcgggacaacgcggaattcaaggaccgcccgcaggacttcaaccgctatcttgactatatgcacggccaggtgaaggagctgctaaccaactatgggacgatcgacgtgctctggttcgatttctct**tac**gaggatatgaccggggaaaaatggaaggcgaccgagctggtgaagatgattcgcgagttgcagccgaatgtgttgattgacaatcggttgggcggcaacatcaaggcccgcgagccggaaatctatgcgggcgacttcgct**tcg**ccggagcagctgcttccgccgcatgggatcgtcaacgaagacgggaagccgcttccgtgggaggcgtgcattacgctgaatcatcattggggctaccacgcgcatgaccgcgactacaagacaccgaagcaggtggtccgcggcctcgtcgaatgcgtgagcaagaacgggaacatg**ctg**ttgaatgtcgggccgaatgcgaagggcgagataccgcagctatcgcttgacgtgctgggcgaagtcggagcctggatgcgcgcgaacggcgacagcatttacggctgtggagcagctgcgttgagcaagccggaatggggacggtacacgcaaaaaggcaataagctgtacgctcatattttggaccggggaatcgggccgatcgcgctgcaaggcttgaatggacgcgtgaaggaagcgcgcttgctcgccgacggagccgaggtcaacattcagacgccgtggaatgcggtcgactacccggattatctgttcgtcaatattccaacggcccagttgccggacgacttcaataccgttatcgagctaacgctggaggatgccgag

**Figure S2.** Nucleotide sequence of the gene encoding α-l-fucosidase iso1 from *Paenibacillus thiaminolyticus*. The gene comprises a single open reading frame of 1278 base pairs, corresponding to a protein of 426 amino acids. Mutated codons are highlighted in bold and shown in different colours.The codon **tac** encodes tyrosine at position 189, **tcg** encodes serine at position 237, and **ctg** encodes leucine at position 297. The following point mutations were introduced: mutant **Y189F** – **tac → ttc**; mutant **S237A** – **tcg → ggc**, mutant **S237G** – **tcg → gcc**, mutant **S237P** – **tcg → cgg**, mutant **S237V** – **tcg → cac**; mutant **L297P** – **ctg → cgg**.

**Table S1.** Nucleotide sequences of primers used for the preparation of plasmids containing genes encoding for potential α-l-transfucosidases.

| **Primer name** | **Nucleotide sequence** |
| --- | --- |
| **Y189F fw** | ATTTCTCT**TTC**GAGGATATGACCGG |
| **Y189F re** | TCATATCCTC**GAA**AGAGAAATCGAACC |
| **S237A fw** | AGCTGCTCCGG**GGC**AGCGAAGTCG |
| **S237A re** | GCT**GCC**CCGGAGCAGCTGCTTCCG |
| **S237G fw** | AGCTGCTCCGG**GCC**AGCGAAGTCG |
| **S237G re** | GCT**GGC**CCGGAGCAGCTGCTTCCG |
| **S237P fw** | AGCTGCTCCGG**CGG**AGCGAAGTCG |
| **S237P re** | GCT**CCG**CCGGAGCAGCTGCTTCCG |
| **S237V fw** | AGCTGCTCCGG**CAC**AGCGAAGTCG |
| **S237V re** | GCT**GTG**CCGGAGCAGCTGCTTCCG |
| **L297P fw** | ATTCAA**CGG**CATGTTCCCGTTCTTGC |
| **L297P re** | ACATG**CCG**TTGAATGTCGGGCCGAATG |

In nucleotide sequences, mutated sites are marked in bold and underlined, with coloured backgrounds highlighting the corresponding mutated codons, as shown in Figure S1.


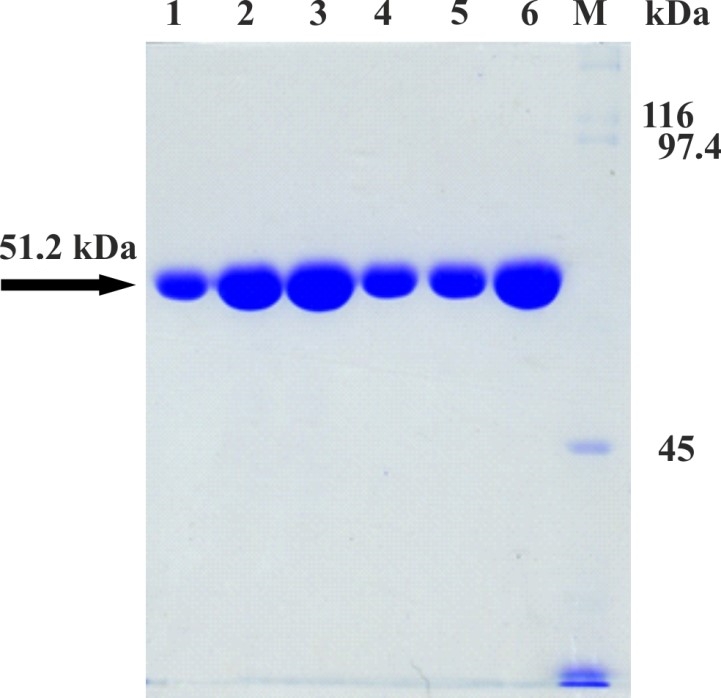


**Figure S3.** Gel after SDS-PAGE of purified α-l-f1*Pth*-wt and its mutated variants produced in *E. coli* BL21(DE3) cells. Lane 1 – α-l-f1*Pth*-wt, lane 2 – mutant S237A, lane 3 – mutant S237G, lane 4 – mutant S237P, lane 5 – mutant S237V, lane 6 – mutant Y189F, lane M – molecular weight standards for SDS-PAGE, Broad Range Standards. All proteins are expressed as fusion constructs with a His-tag, resulting in a uniform molecular weight of 51.2 kDa. The arrow indicates the position of the purified proteins on the gel.


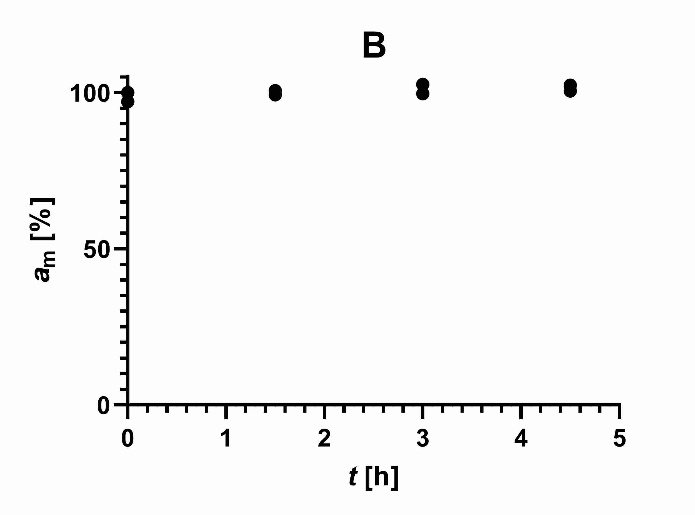

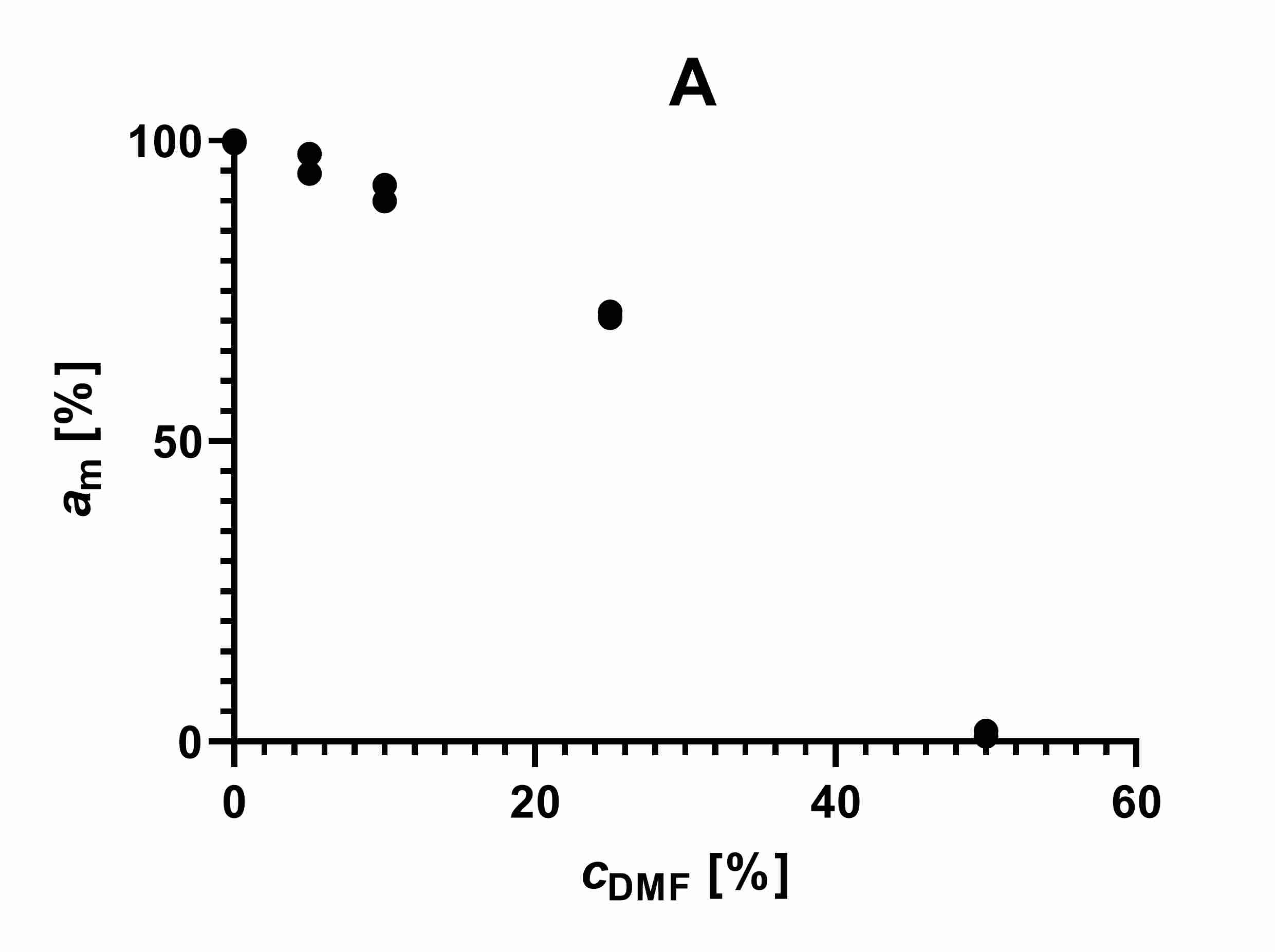


**Figure S4.** (**A**) Dependence of α-l-f1*Pth*-wt activity on the concentration of DMF in the reaction and (**B**) stability of α-l-f1*Pth*-wt after incubation with 10% DMF (*v/v*). **A** – All activity measurements were performed in 25 mM EPPS buffer (pH 8.0) at 37 °C for 10 minutes using 7 mM *p*NP-α-l-Fuc as substrate. The initial concentrations of DMF in the reaction mixtures were 0, 5, 10, 25 and 50% (*v*/*v*). To stop the reaction, 200 µL of 10% (*v*/*v*) Na_2_CO_3_ was added to 200 µL of the reaction mixture. The amount of released *p*-nitrophenol was determined by measuring the absorbance increase at 405 nm. The obtained absorbance values were converted to relative enzyme activity (*a*_m_). **B** – α-l-f1*Pth*-wt was first incubated in the presence of 10% DMF (*v*/*v*) for 1.5, 3 and 4.5 h at 37 °C. After incubation, the activity assay was performed as described for **A**, with the DMF concentration fixed at 10% (*v*/*v*).

1. **Structural characterization of prepared transfucosylation products**

# Table S2. ^1^H and ^13^C NMR data of compound 3 (700.13 MHz for ^1^H, 176.05 MHz for ^13^C, D_2_O, 30 °C)

|  | **Atom** | ***δ*_C_** | **m.** | ***δ*_H_** | **n_H_** | **m.** | ***J* [Hz]** | **diagnostic HMBC** |
| --- | --- | --- | --- | --- | --- | --- | --- | --- |
| **Boc** | **CO** | 158.49 | S | - | 0 | - |  | 2**’** |
|  | **C** | 81.32 | S | - | 0 | - |  | (CH_3_)_3_ |
|  | **(CH_3_)_3_** | 27.87 | Q | 1.424 | 9 | s |  |  |
| **spacer** | **1’** | 44.52 | T | 3.69^H^ | 2 | br m |  |  |
|  | **2’** | 39.46 | T | 3.288 | 2 | br t |  |  |
|  | **CS** | 183.30^a^ | S | - | 0 | - |  |  |
| **Glc^A^** | **1** | 83.23^a^ | D | 5.60, 5.33^a^ | 1 | br s |  |  |
|  | **2** | 71.88 | D | 3.490 | 1 | br dd |  |  |
|  | **3** | 75.30 | D | 3.71^H^ | 1 | m |  |  |
|  | **4** | 78.05 | D | 3.70^H^ | 1 | m |  | 1^B^ |
|  | **5** | 76.14 | D | 3.68^H^ | 1 | m |  |  |
|  | **6** | 60.09 | T | 3.944 | 1 | dd |  |  |
|  |  |  |  | 3.823 | 1 | dd | 12.3, 4.2 |  |
| **Gal^B^** | **1** | 102.87 | D | 4.519 | 1 | d | 7.2 |  |
|  | **2** | 70.59 | D | 3.720 | 1 | m |  |  |
|  | **3** | 80.50 | D | 3.696 | 1 | m |  | 1^C^ |
|  | **4** | 68.82 | D | 4.009 | 1 | dd | 2.9, 0.5 |  |
|  | **5** | 75.46 | D | 3.74^H^ | 1 | m |  |  |
|  | **6** | 61.13 | T | 3.78^H^ | 1 | m |  |  |
|  |  |  |  | 3.74^H^ | 1 | m |  |  |
| **Fuc^C^** | **1** | 101.08 | D | 5.167 | 1 | d | 4.1 | 3^B^ |
|  | **2** | 68.61 | D | 3.774 | 1 | dd | 10.5, 4.1 |  |
|  | **3** | 69.59 | D | 3.930 | 1 | dd | 10.5, 3.4 |  |
|  | **4** | 71.95 | D | 3.820 | 1 | dd | 3.4, 0.9 |  |
|  | **5** | 67.35 | D | 4.164 | 1 | dq | 0.9, 6.7 |  |
|  | **6** | 15.51 | T | 1.201 | 3 | d | 6.7 |  |

^a^ … tentative assignment; ^H^ … HSQC readout

**
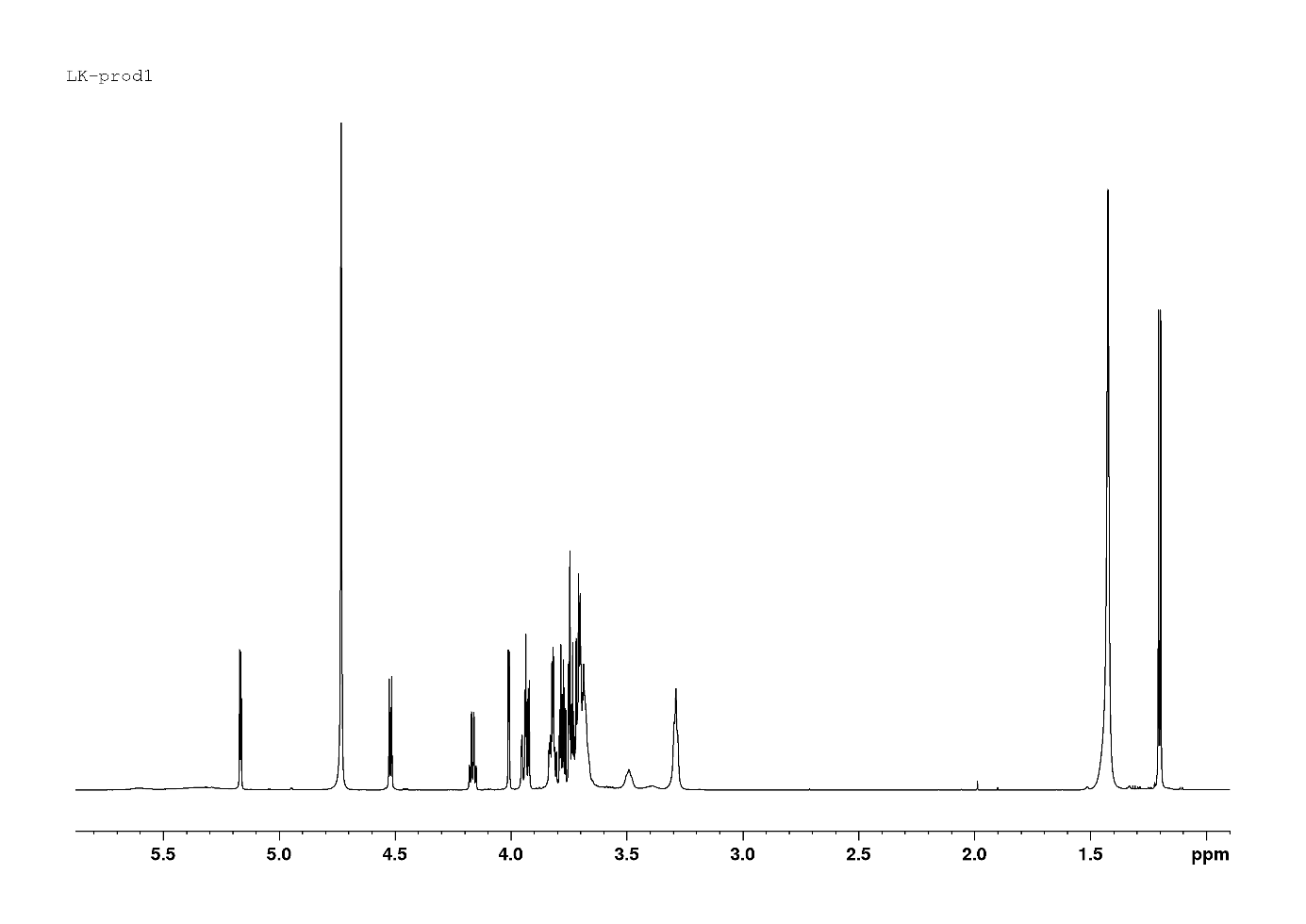
**

Figure S5A. ^1^H NMR spectrum of compound **3** (700.13 MHz for ^1^H, D_2_O, 30 °C).


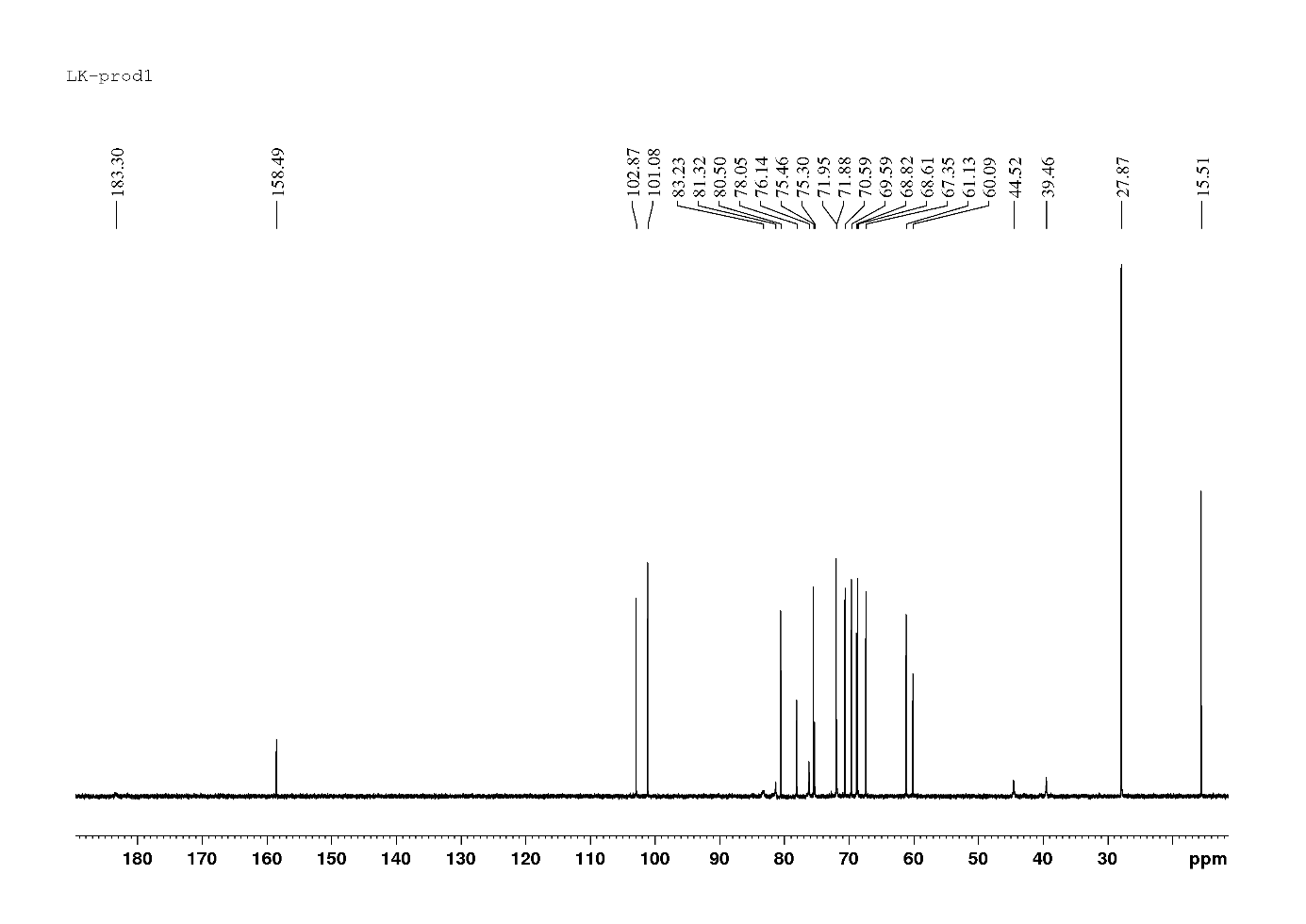


**Figure S5B.** ^13^C NMR spectrum of compound **3** (176.05 MHz for ^13^C, D_2_O, 30 °C).

#
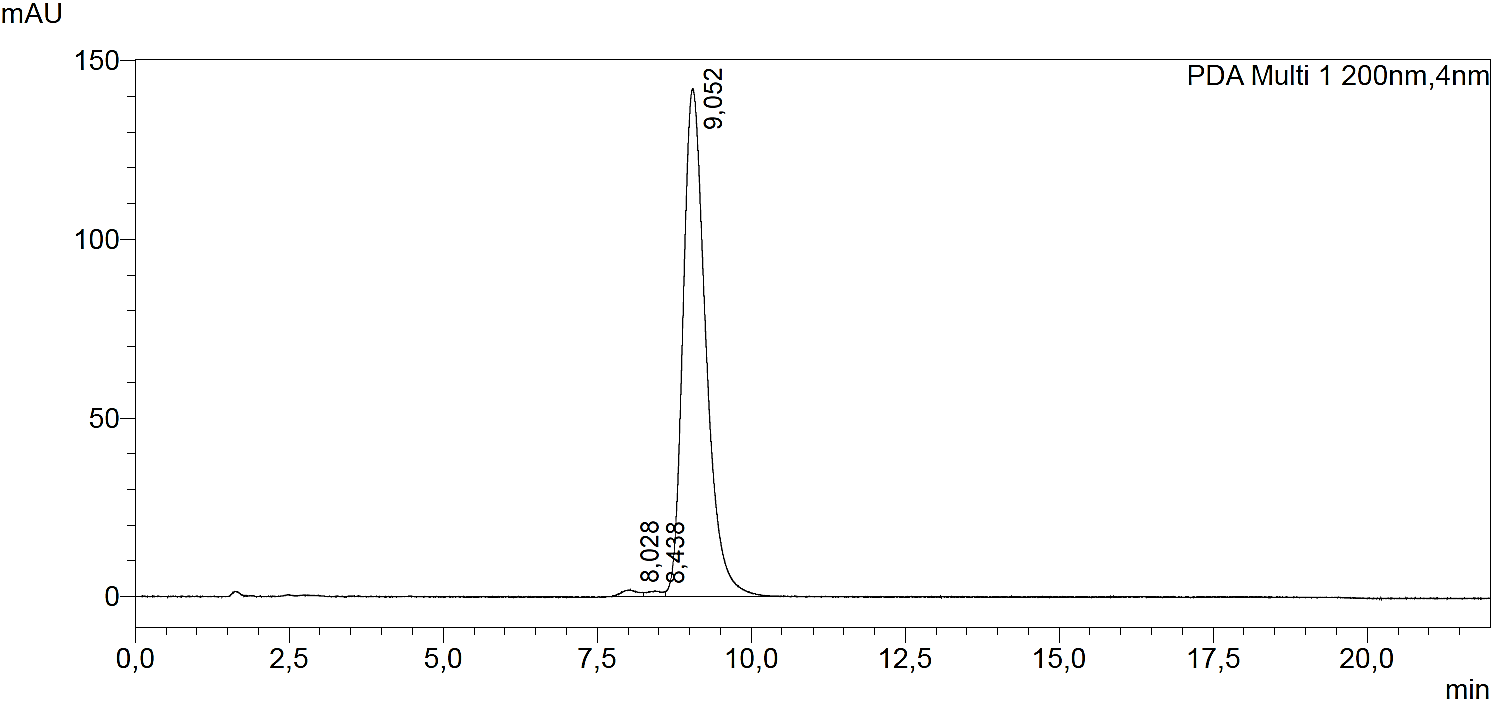


# Figure S5C. HPLC chromatogram of compound 3 (9.052 min; purity 98%).


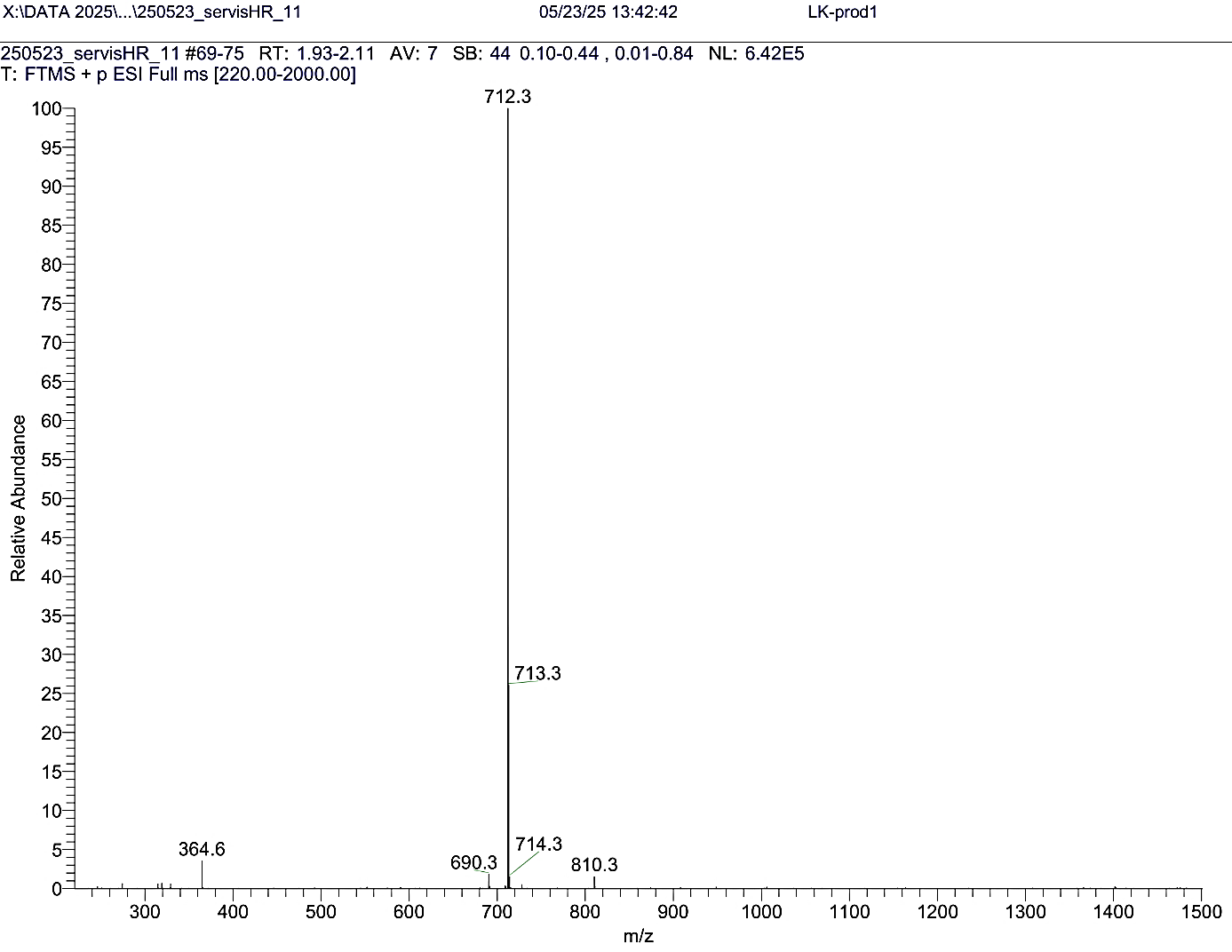
**Figure S5D.** MS spectrum (ESI^+^) of compound **3**: [M + H]^+^, *m*/*z* 690.3; [M + Na]^+^, *m*/*z* 712.3. HRMS (ESI^+^): *m*/*z* for C_26_H_48_O_16_N_3_S calculated 690.27498, found 690.27467 (-0.44271 ppm); *m*/*z* for C_26_H_47_O_16_N_3_NaS calculated 712.25692, found 712.25671 (-0.30679 ppm).

# Table S3. ^1^H and ^13^C NMR data of compound 4 (700.13 MHz for ^1^H, 176.05 MHz for ^13^C, D_2_O, 30 °C)

|  | **Atom** | ***δ*_C_** | **m.** | ***δ*_H_** | **n_H_** | **m.** | ***J* [Hz]** | **diagnostic HMBC** |
| --- | --- | --- | --- | --- | --- | --- | --- | --- |
| **Boc** | **CO** | 158.49 | S | - | 0 | - |  | 2**’** |
|  | **C** | 81.32 | S | - | 0 | - |  | (CH_3_)_3_ |
|  | **(CH_3_)_3_** | 27.87 | Q | 1.423 | 9 | s |  |  |
| **spacer** | **1’** | 44.50 | T | 3.68^H^ | 2 | br m |  |  |
|  | **2’** | 39.47 | T | 3.287 | 2 | br t |  |  |
|  | **CS** | 183.35^a^ | S | - | 0 | - |  |  |
| **Glc^A^** | **1** | 83.15^a^ | D | 5.60, 5.33^a^ | 1 | br s |  |  |
|  | **2** | 71.89 | D | 3.469 | 1 | br dd |  |  |
|  | **3** | 75.31 | D | 3.718 | 1 | br dd |  |  |
|  | **4** | 78.94 | D | 3.645 | 1 | br dd |  | 1^B^ |
|  | **5** | 75.92 | D | 3.688 | 1 | ddd | 9.8, 4.3, 2.0 |  |
|  | **6** | 60.06 | T | 3.928 | 1 | dd | 12.3, 2.0 |  |
|  |  |  |  | 3.821 | 1 | dd | 12.3, 4.3 |  |
| **Gal^B^** | **1** | 103.34 | D | 4.451 | 1 | d | 7.9 | 4^A^ |
|  | **2** | 70.92 | D | 3.572 | 1 | dd | 9.9, 7.9 |  |
|  | **3** | 72.71 | D | 3.669 | 1 | dd | 9.9, 3.4 |  |
|  | **4** | 68.72 | D | 3.949 | 1 | dd | 3.4, 0.7 |  |
|  | **5** | 73.94 | D | 3.918 | 1 | m |  |  |
|  | **6** | 67.54 | T | 3.867 | 1 | dd | 10.9, 4.1 | 1^C^ |
|  |  |  |  | 3.773 | 1 | dd |  |  |
| **Fuc^C^** | **1** | 99.45 | D | 4.945 | 1 | d | 3.9 | 6^B^ |
|  | **2** | 68.40 | D | 3.766 | 1 | dd | 10.4, 3.9 |  |
|  | **3** | 69.73 | D | 3.884 | 1 | dd | 10.4, 3.4 |  |
|  | **4** | 71.99 | D | 3.802 | 1 | dd | 3.4, 0.9 |  |
|  | **5** | 66.97 | D | 4.091 | 1 | dq | 0.9, 6.7 |  |
|  | **6** | 15.50 | T | 1.217 | 3 | d | 6.7 |  |

^a^ … tentative assignment; ^H^ … HSQC readout


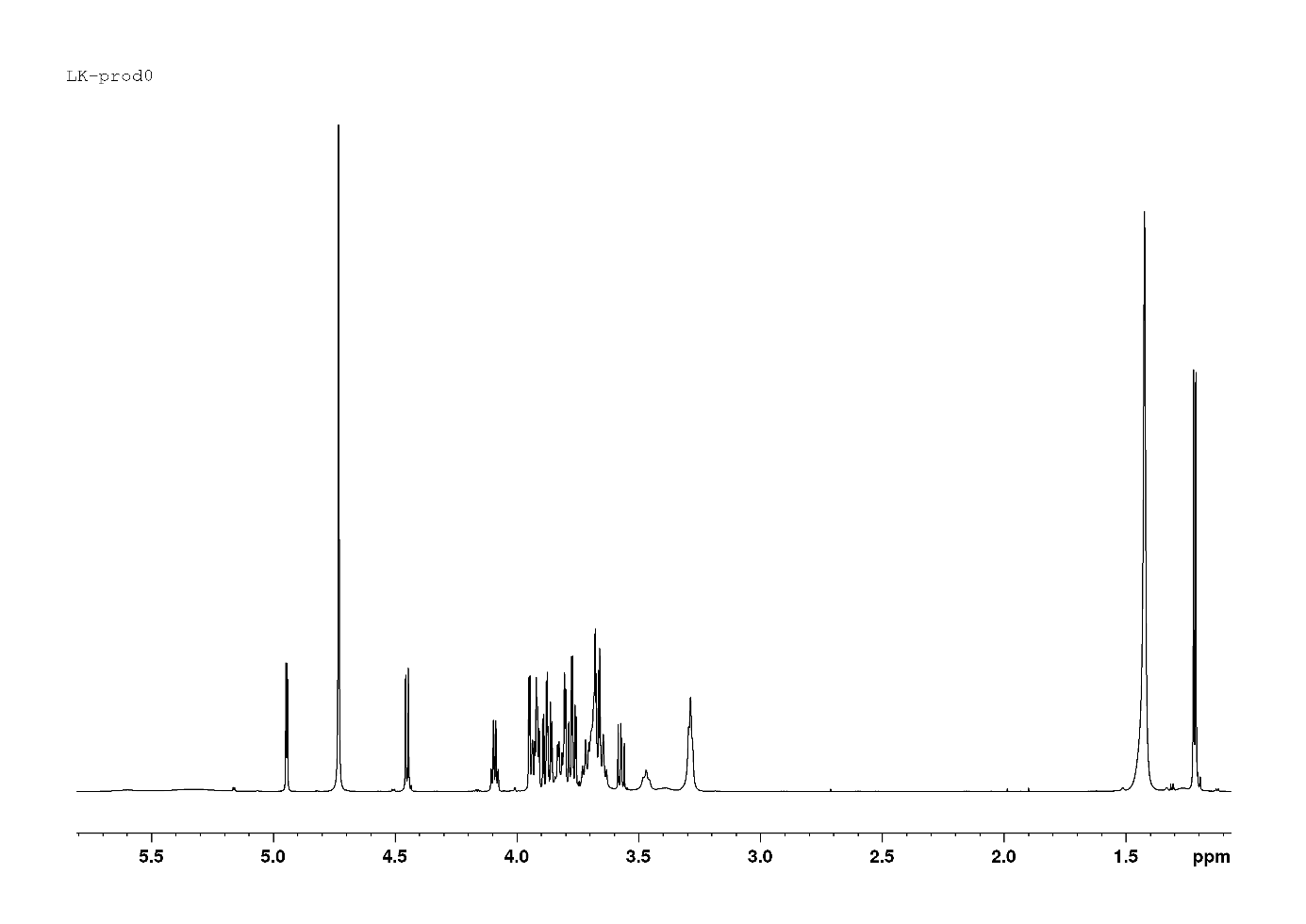


Figure S6A. ^1^H NMR spectrum of compound **4** (700.13 MHz for ^1^H, D_2_O, 30 °C).


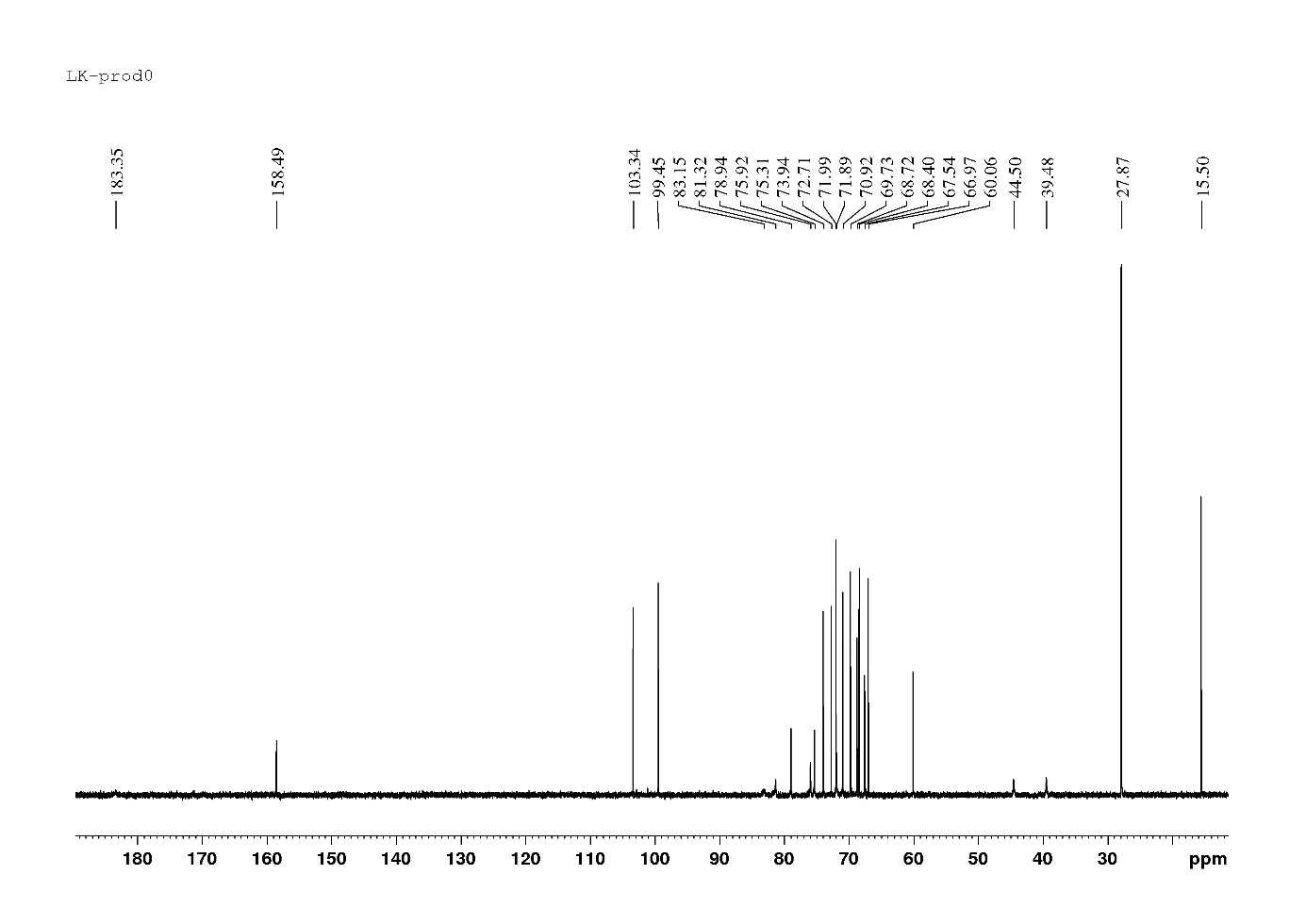


**Figure S6B.** ^13^C NMR spectrum of compound **4** (176.05 MHz for ^13^C, D_2_O, 30 °C).


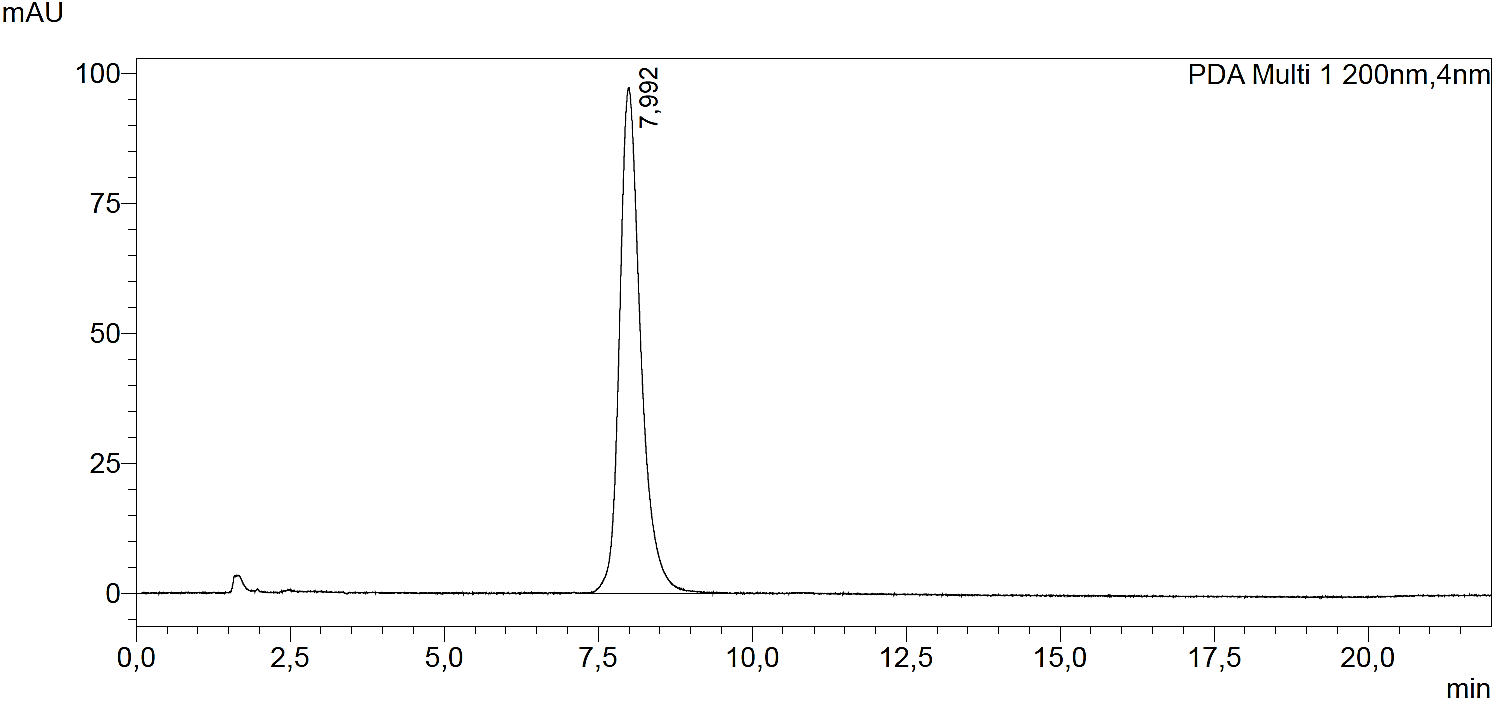


# Figure S6C. HPLC chromatogram of compound 4 (7.992 min; purity 99%).


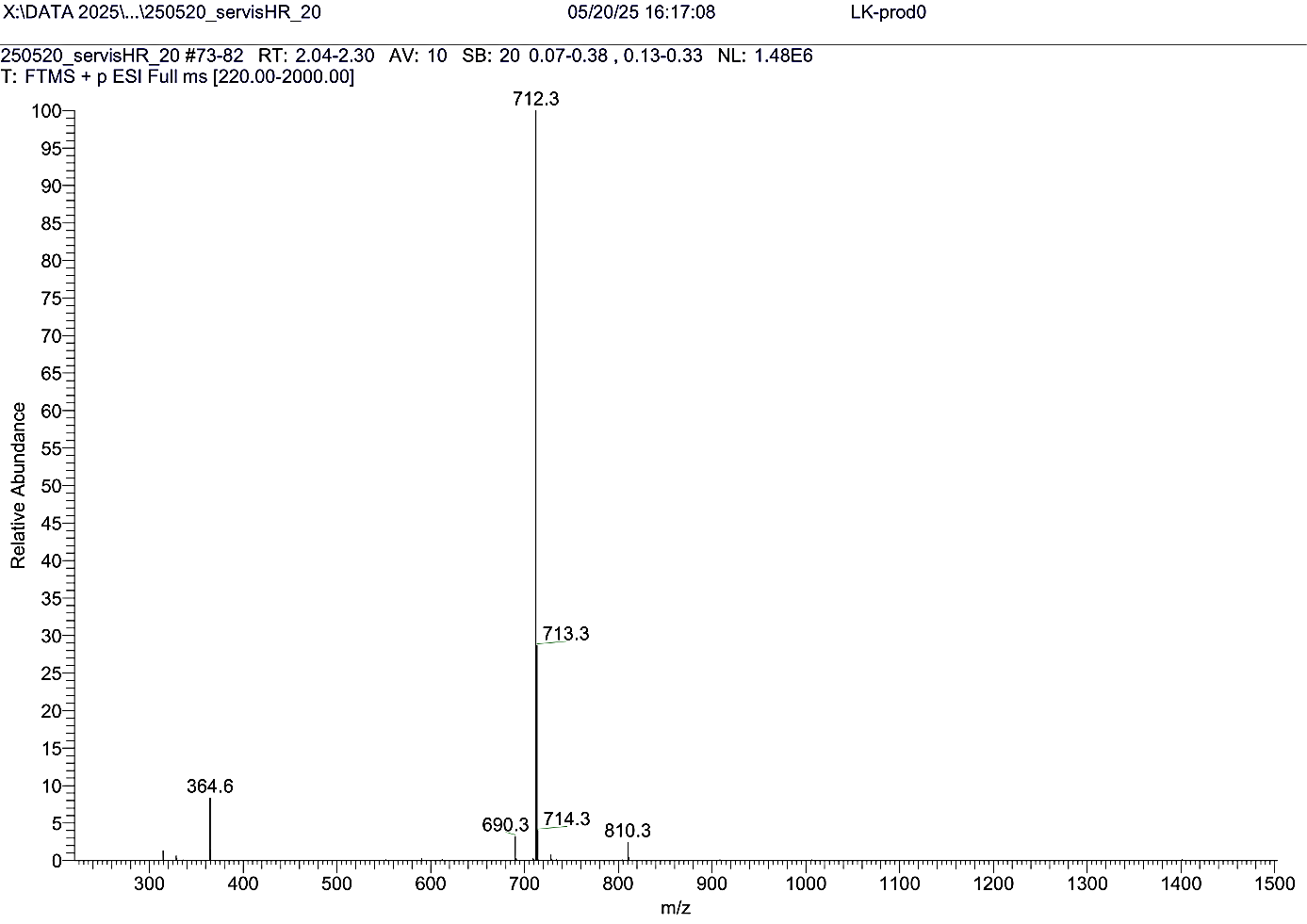
**Figure S6D.** MS spectrum (ESI^+^) of compound **4**: [M + H]^+^, *m*/*z* 690.3; [M + Na]^+^, *m*/*z* 712.3. HRMS (ESI^+^): *m*/*z* for C_26_H_48_O_16_N_3_S calculated 690.27498, found 690.27467 (-0.44842 ppm); *m*/*z* for C_26_H_47_O_16_N_3_NaS calculated 712.25692, found 712.25663 (-0.41073 ppm).

**Table S4.** ^1^H and ^13^C NMR data of compound **5** (700.13 MHz for ^1^H, 176.05 MHz for ^13^C, D_2_O, 30 °C)

|  | **Atom** | ***δ*_C_** | **m.** | ***δ*_H_** | **n_H_** | **m.** | ***J* [Hz]** | **diagnostic HMBC** |
| --- | --- | --- | --- | --- | --- | --- | --- | --- |
| **Boc** | **CO** | 158.49 | S | - | 0 | - |  | 2**’** |
|  | **C** | 81.32 | S | - | 0 | - |  | (CH_3_)_3_ |
|  | **(CH_3_)_3_** | 27.87 | Q | 1.425 | 9 | s |  |  |
| **spacer** | **1’** | 44.46 | T | 3.68^H^ | 2 | br m |  |  |
|  | **2’** | 39.47 | T | 3.286 | 2 | br t |  |  |
|  | **CS** | 183.67^a^ | S | - | 0 | - |  |  |
| **Glc^A^** | **1** | 83.48^a^ | D | 5.58, 5.36^a^ | 1 | br s |  |  |
|  | **2** | 71.89 | D | 3.502 | 1 | br dd |  |  |
|  | **3** | 75.28 | D | 3.70^H^ | 1 | m |  |  |
|  | **4** | 78.04 | D | 3.76^H^ | 1 | m |  | 1^B^ |
|  | **5** | 75.36 | D | 3.75^H^ | 1 | m |  |  |
|  | **6** | 67.28 | T | 4.012 | 1 | br d | 11.8 | 1^C^ |
|  |  |  |  | 3.845 | 1 | dm | 11.8 |  |
| **Gal^B^** | **1** | 102.96 | D | 4.505 | 1 | d | 7.8 |  |
|  | **2** | 71.10 | D | 3.584 | 1 | dd | 9.9, 7.8 |  |
|  | **3** | 72.72 | D | 3.651 | 1 | dd | 9.9, 3.4 |  |
|  | **4** | 68.81 | D | 3.917 | 1 | dd | 3.4, 0.7 |  |
|  | **5** | 75.52 | D | 3.702 | 1 | ddd | 9.1, 3.9, 0.7 |  |
|  | **6** | 61.28 | T | 3.789 | 1 | dd | 11.8, 8.1 |  |
|  |  |  |  | 3.747 | 1 | dd | 11.8, 3.9 |  |
| **Fuc^C^** | **1** | 99.75 | D | 4.917 | 1 | d | 3.9 | 6^A^ |
|  | **2** | 68.37 | D | 3.761 | 1 | dd | 10.4, 3.9 |  |
|  | **3** | 69.67 | D | 3.880 | 1 | dd | 10.4, 3.4 |  |
|  | **4** | 72.06 | D | 3.775 | 1 | br dd | 3.4, 0.9 |  |
|  | **5** | 66.98 | D | 4.200 | 1 | br q |  |  |
|  | **6** | 15.51 | T | 1.199 | 3 | d | 6.6 |  |

^a^ … tentative assignment; ^H^ … HSQC readout


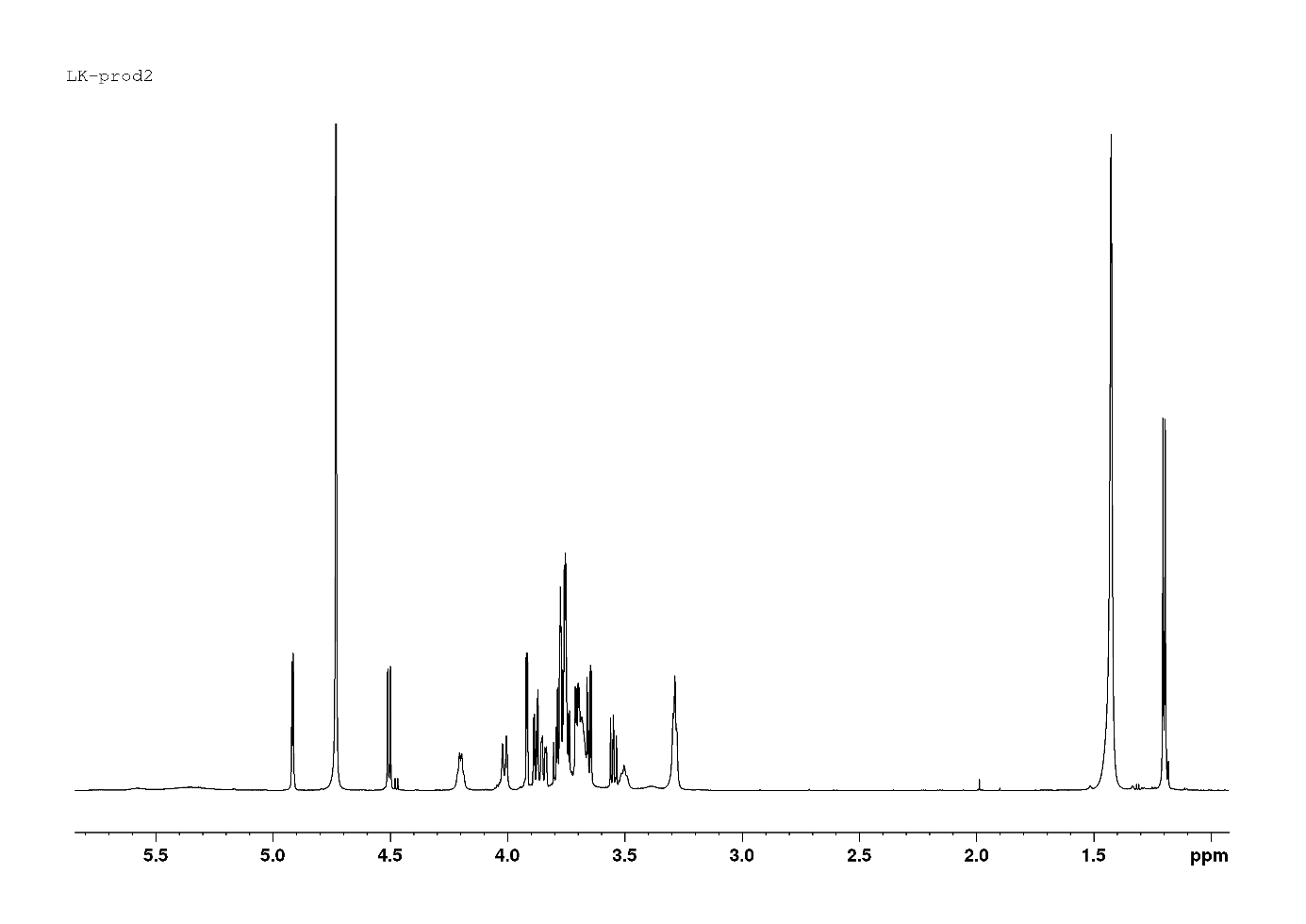


**Figure S7A.** ^1^H NMR spectrum of compound **5** (700.13 MHz for ^1^H, D_2_O, 30 °C).

**
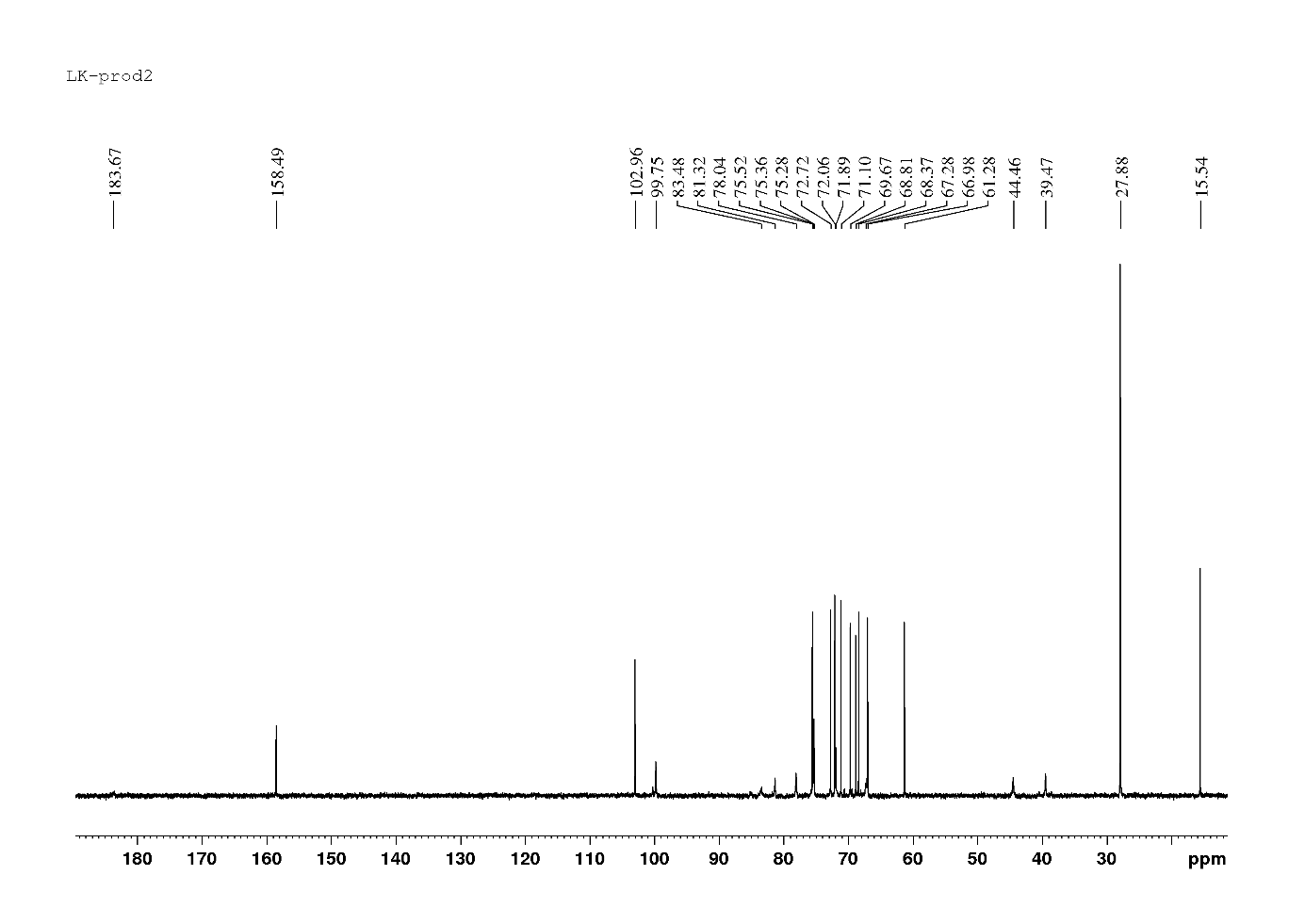
**

**Figure S7B.** ^13^C NMR spectrum of compound **5** (176.05 MHz for ^13^C, D_2_O, 30 °C).


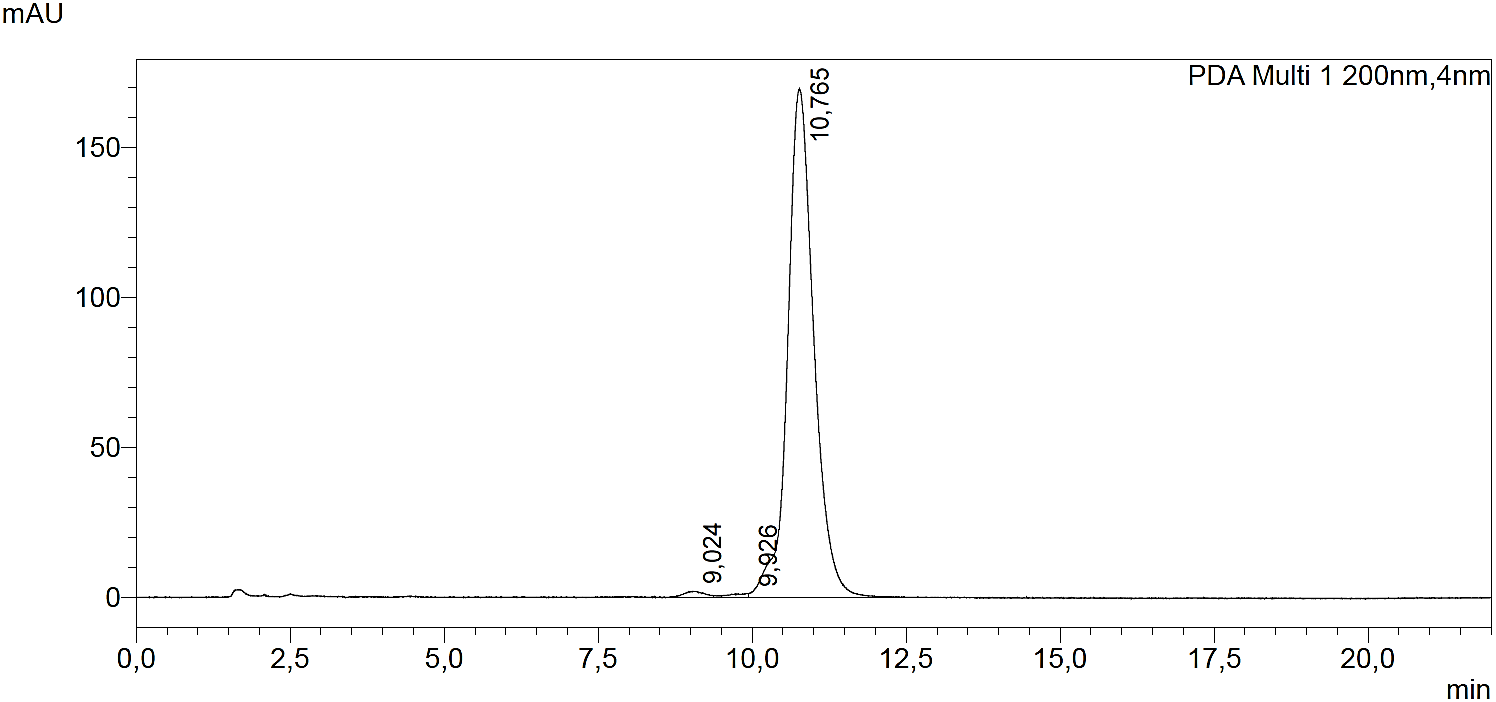


**Figure S7C.** HPLC chromatogram of compound **5** (10.765 min; purity 99%).


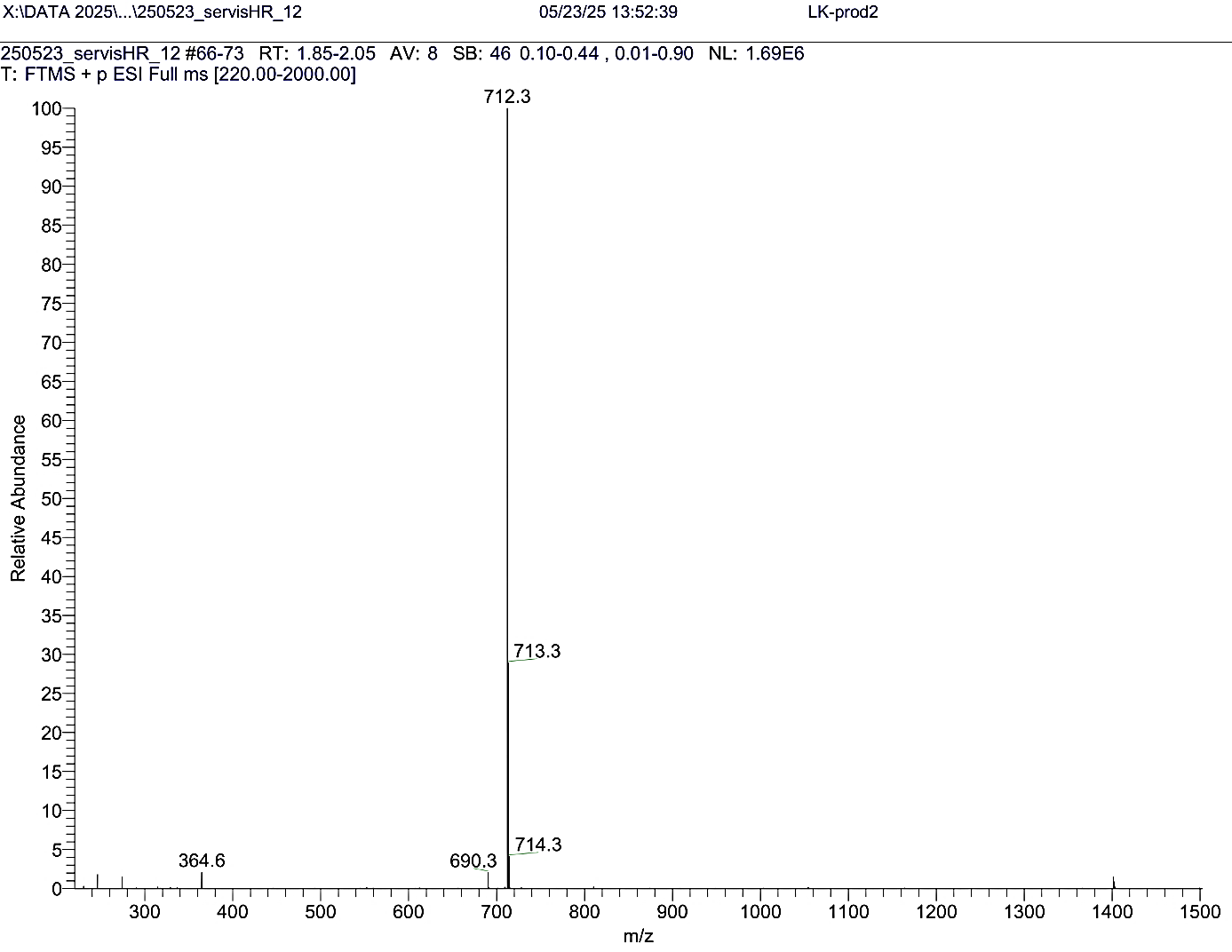


**Figure S7D.** MS spectrum (ESI+) of compound **5**: [M + H]^+^, *m*/*z* 690.3; [M + Na]^+^, *m*/*z* 712.3. HRMS (ESI^+^): *m*/*z* for C_26_H_48_O_16_N_3_S calculated 690.27498, found 690.27458 (-0.57348 ppm); *m*/*z* for C_26_H_47_O_16_N_3_NaS calculated 712.25692, found 712.25643 (-0.68834 ppm).

**Table S5.** ^1^H and ^13^C NMR data of compound **7a** (700.13 MHz for ^1^H, 176.05 MHz for ^13^C, D_2_O, 30 °C). ^H^ stands for HSQC readout.

**α-anomer**

|  | **Atom** | ***δ*_C_** | **m.** | ***δ*_H_** | **n_H_** | **m.** | ***J* [Hz]** | **diagnostic HMBC** |
| --- | --- | --- | --- | --- | --- | --- | --- | --- |
| **Glc^A^** | **1** | 92.03 | D | 5.228 | 1 | d | 3.9 |  |
|  | **2** | 71.36 | D | 3.588 | 1 | dd | 9.9, 3.9 |  |
|  | **3** | 71.65 | D | 3.843 | 1 | dd | 9.9, 8.8 |  |
|  | **4** | 78.71 | D | 3.669 | 1 | dd | 10.0, 8.8 | 1^B^ |
|  | **5** | 70.30 | D | 3.957 | 1 | ddd | 10.0, 2.2, 4.5 |  |
|  | **6** | 60.18 | T | 3.90^H^ | 1 | m |  |  |
|  |  |  |  | 3.85^H^ | 1 | m |  |  |
| **Gal^B^** | **1** | 102.90 | D | 4.515 | 1 | d | 7.4 |  |
|  | **2** | 70.63 | D | 3.71^H^ | 1 | m |  |  |
|  | **3** | 80.54 | D | 3.71^H^ | 1 | m |  | 1^C^ |
|  | **4** | 68.84 | D | 4.019 | 1 | br d | 2.7 |  |
|  | **5** | 75.46 | D | 3.75^H^ | 1 | m |  |  |
|  | **6** | 61.13 | T | 3.79^H^ | 1 | m |  |  |
|  |  |  |  | 3.74^H^ | 1 | m |  |  |
| **Fuc^C^** | **1** | 101.10 | D | 5.176 | 1 | d | 4.1 | 3^B^ |
|  | **2** | 68.63 | D | 3.786 | 1 | dd | 10.4, 4.1 |  |
|  | **3** | 69.62 | D | 3.942 | 1 | dd | 10.4, 3.4 |  |
|  | **4** | 71.97 | D | 3.831 | 1 | br d | 3.4 |  |
|  | **5** | 67.37 | D | 4.174 | 1 | br q | 6.6 |  |
|  | **6** | 15.52 | T | 1.212 | 3 | d | 6.6 |  |

**β-anomer**

|  | **Atom** | ***δ*_C_** | **m.** | ***δ*_H_** | **n_H_** | **m.** | ***J* [Hz]** | **diagnostic HMBC** |
| --- | --- | --- | --- | --- | --- | --- | --- | --- |
| **Glc^A^** | **1** | 95.98 | D | 4.669 | 1 | d | 8.0 |  |
|  | **2** | 74.03 | D | 3.292 | 1 | dd | 8.9, 8.0 |  |
|  | **3** | 74.60 | D | 3.648 | 1 | dd | 8.9, 8.9 |  |
|  | **4** | 78.58 | D | 3.681 | 1 | dd | 9.3, 8.9 | 1^B^ |
|  | **5** | 74.99 | D | 3.604 | 1 | ddd | 9.3, 5.1, 2.2 |  |
|  | **6** | 60.31 | T | 3.96^H^ | 1 | m |  |  |
|  |  |  |  | 3.81^H^ | 1 | m |  |  |
| **Gal^B^** | **1** | 102.92 | D | 4.515 | 1 | d | 7.4 |  |
|  | **2** | 70.62 | D | 3.73^H^ | 1 | m |  |  |
|  | **3** | 80.54 | D | 3.71^H^ | 1 | m |  | 1^C^ |
|  | **4** | 68.82 | D | 4.019 | 1 | br d | 2.7 |  |
|  | **5** | 75.46 | D | 3.75^H^ | 1 | m |  |  |
|  | **6** | 61.12 | T | 3.79^H^ | 1 | m |  |  |
|  |  |  |  | 3.74^H^ | 1 | m |  |  |
| **Fuc^C^** | **1** | 101.10 | D | 5.176 | 1 | d | 4.1 | 3^B^ |
|  | **2** | 68.63 | D | 3.786 | 1 | dd | 10.4, 4.1 |  |
|  | **3** | 69.62 | D | 3.940 | 1 | dd | 10.4, 3.4 |  |
|  | **4** | 71.97 | D | 3.831 | 1 | br d | 3.4 |  |
|  | **5** | 67.37 | D | 4.174 | 1 | br q | 6.6 |  |
|  | **6** | 15.52 | T | 1.212 | 3 | d | 6.6 |  |

**Table S6.** ^1^H and ^13^C NMR data of compound **7b** (700.13 MHz for ^1^H, 176.05 MHz for ^13^C, D_2_O, 30 °C). ^H^ stands for HSQC readout.

**α-anomer**

|  | **Atom** | ***δ*_C_** | **m.** | ***δ*_H_** | **n_H_** | **m.** | ***J* [Hz]** | **diagnostic HMBC** |
| --- | --- | --- | --- | --- | --- | --- | --- | --- |
| **Glc^A^** | **1** | 92.03 | D | 5.223 | 1 | d | 3.9 |  |
|  | **2** | 71.39 | D | 3.564 | 1 | dd | 9.9, 3.9 |  |
|  | **3** | 71.70 | D | 3.850 | 1 | dd | 9.9, 8.7 |  |
|  | **4** | 79.69 | D | 3.614 | 1 | dd |  | 1^B^ |
|  | **5** | 70.08 | D | 3.96^H^ | 1 | m |  |  |
|  | **6** | 60.15 | T | 3.88^H^ | 1 | m |  |  |
|  |  |  |  | 3.86^H^ | 1 | m |  |  |
| **Gal^B^** | **1** | 103.39 | D | 4.449 | 1 | d | 7.8 |  |
|  | **2** | 70.94 | D | 3.581 | 1 | dd | 9.9, 7.8 |  |
|  | **3** | 72.75 | D | 3.683 | 1 | dd | 9.9, 3.4 |  |
|  | **4** | 68.74 | D | 3.959 | 1 | br d | 3.4 |  |
|  | **5** | 73.95 | D | 3.92^H^ | 1 | m |  |  |
|  | **6** | 67.63 | T | 3.88^H^ | 1 | m |  | 1^C^ |
|  |  |  |  | 3.79^H^ | 1 | m |  |  |
| **Fuc^C^** | **1** | 99.49 | D | 4.951 | 1 | d | 3.9 | 6^B^ |
|  | **2** | 68.42 | D | 3.774 | 1 | dd | 10.4, 3.9 |  |
|  | **3** | 69.73 | D | 3.897 | 1 | dd | 10.4, 3.4 |  |
|  | **4** | 72.01 | D | 3.813 | 1 | br d | 3.4 |  |
|  | **5** | 66.99 | D | 4.104 | 1 | br q | 6.7 |  |
|  | **6** | 15.52 | T | 1.229 | 3 | d | 6.7 |  |

**β-anomer**

|  | **Atom** | ***δ*_C_** | **m.** | ***δ*_H_** | **n_H_** | **m.** | ***J* [Hz]** | **diagnostic HMBC** |
| --- | --- | --- | --- | --- | --- | --- | --- | --- |
| **Glc^A^** | **1** | 95.86 | D | 4.675 | 1 | d | 8.1 |  |
|  | **2** | 74.05 | D | 3.268 | 1 | dd | 8.9, 8.1 |  |
|  | **3** | 74.63 | D | 3.65^H^ | 1 | m |  |  |
|  | **4** | 79.49 | D | 3.63^H^ | 1 | m |  | 1^B^ |
|  | **5** | 74.78 | D | 3.62^H^ | 1 | m |  |  |
|  | **6** | 60.29 | T | 3.95^H^ | 1 | m |  |  |
|  |  |  |  | 3.81^H^ | 1 | m |  |  |
| **Gal^B^** | **1** | 103.41 | D | 4.449 | 1 | d | 7.8 |  |
|  | **2** | 70.94 | D | 3.572 | 1 | dd | 9.9, 7.8 |  |
|  | **3** | 72.75 | D | 3.679 | 1 | dd | 9.9, 3.4 |  |
|  | **4** | 68.73 | D | 3.959 | 1 | br d | 3.4 |  |
|  | **5** | 73.94 | D | 3.92^H^ | 1 | m |  |  |
|  | **6** | 67.59 | T | 3.88^H^ | 1 | m |  | 1^C^ |
|  |  |  |  | 3.78^H^ | 1 | m |  |  |
| **Fuc^C^** | **1** | 99.48 | D | 4.951 | 1 | d | 3.9 | 6^B^ |
|  | **2** | 68.41 | D | 3.777 | 1 | dd | 10.4, 3.9 |  |
|  | **3** | 69.73 | D | 3.897 | 1 | dd | 10.4, 3.4 |  |
|  | **4** | 72.01 | D | 3.813 | 1 | br d | 3.4 |  |
|  | **5** | 66.99 | D | 4.104 | 1 | br q | 6.7 |  |
|  | **6** | 15.52 | T | 1.229 | 3 | d | 6.7 |  |

**
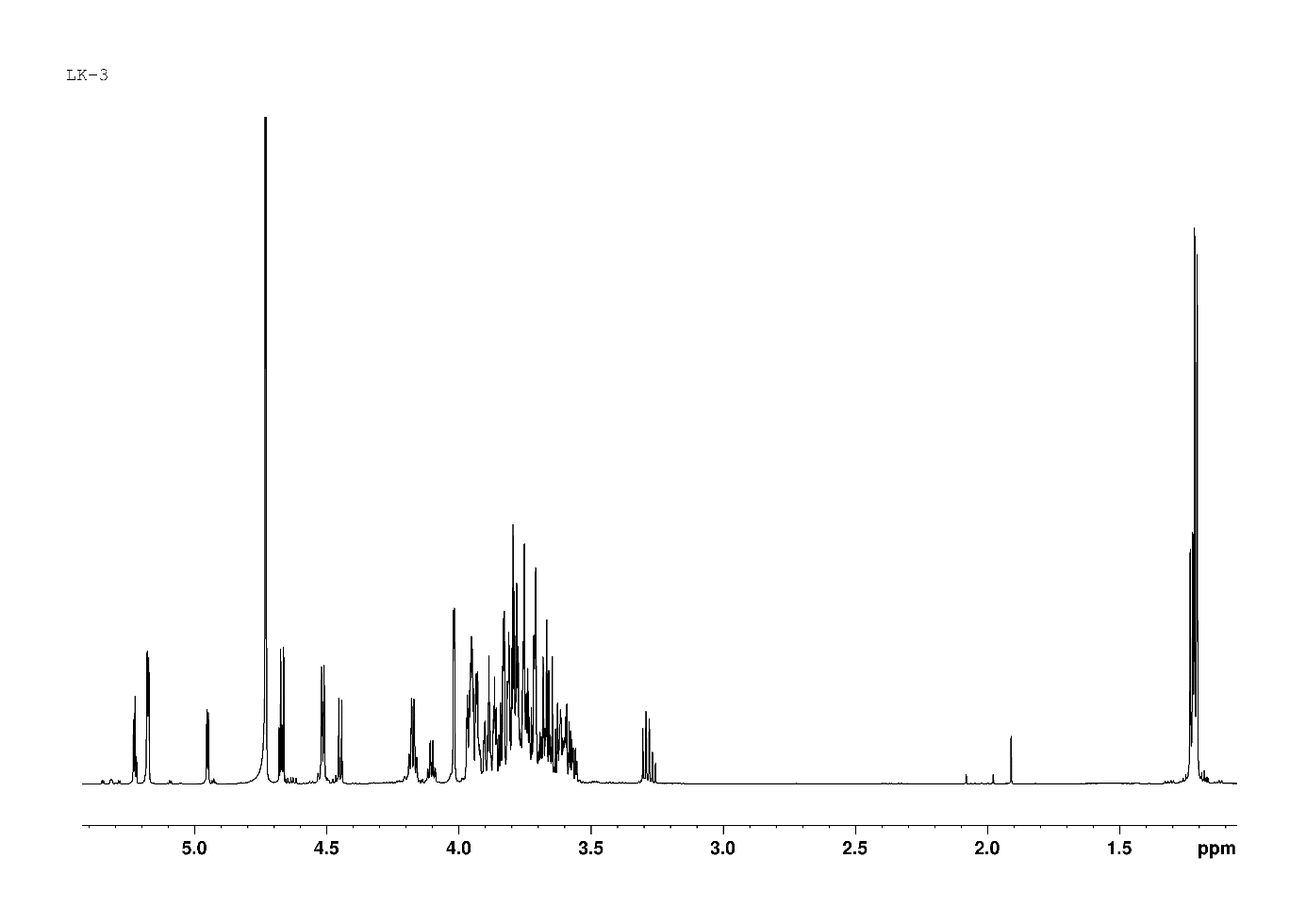
**

**Figure S8A.** ^1^H NMR spectrum of a mixture of compounds **7a** and **7b** (700.13 MHz for ^1^H, D_2_O, 30 °C).


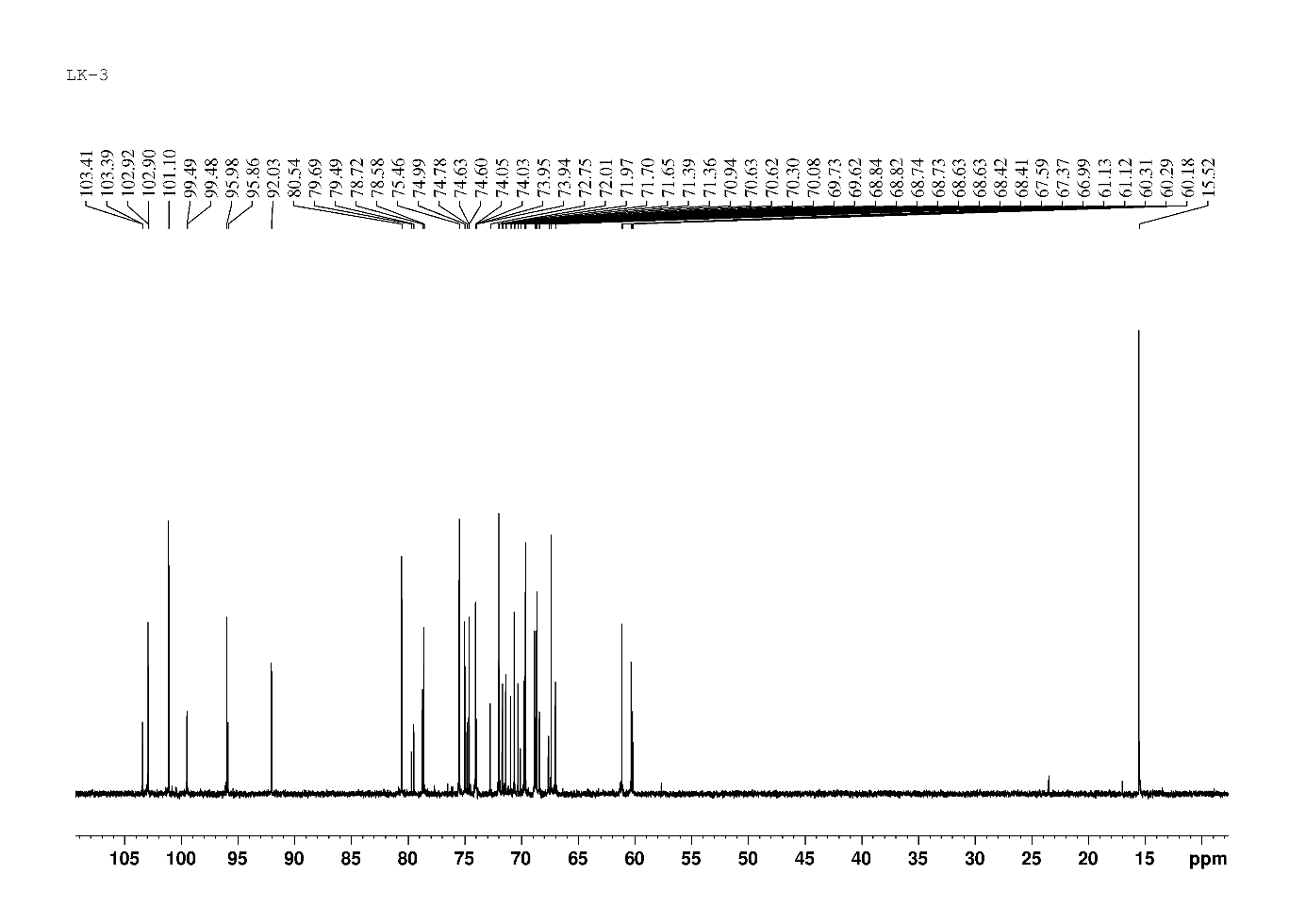
**Figure S8B.** ^13^C NMR spectrum of a mixture of compounds **7a** and **7b** (176.05 MHz for ^13^C, D_2_O, 30 °C).


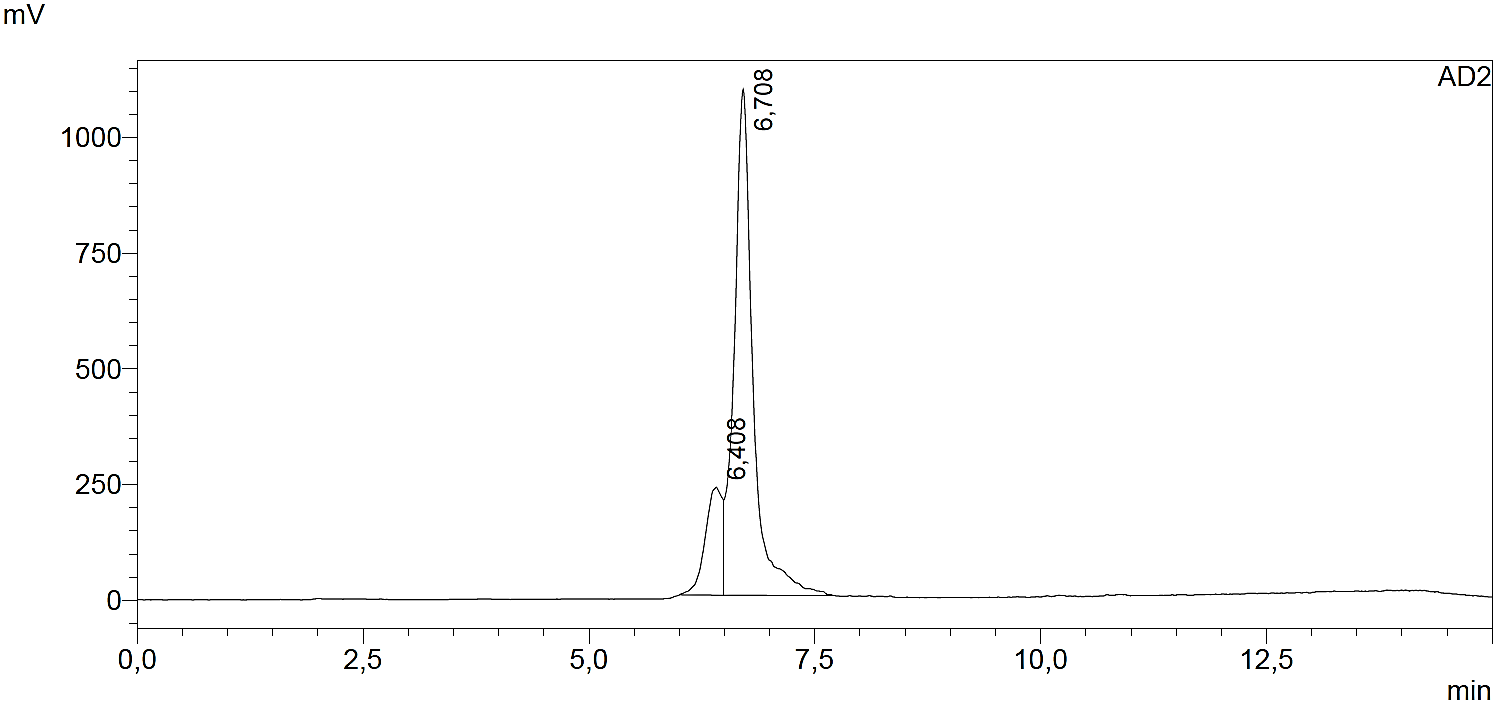


**Figure S8C.** HPLC chromatogram of a mixture of compounds **7a** and **7b** (6.408 and 6.708 min, respectively; purity 99%).


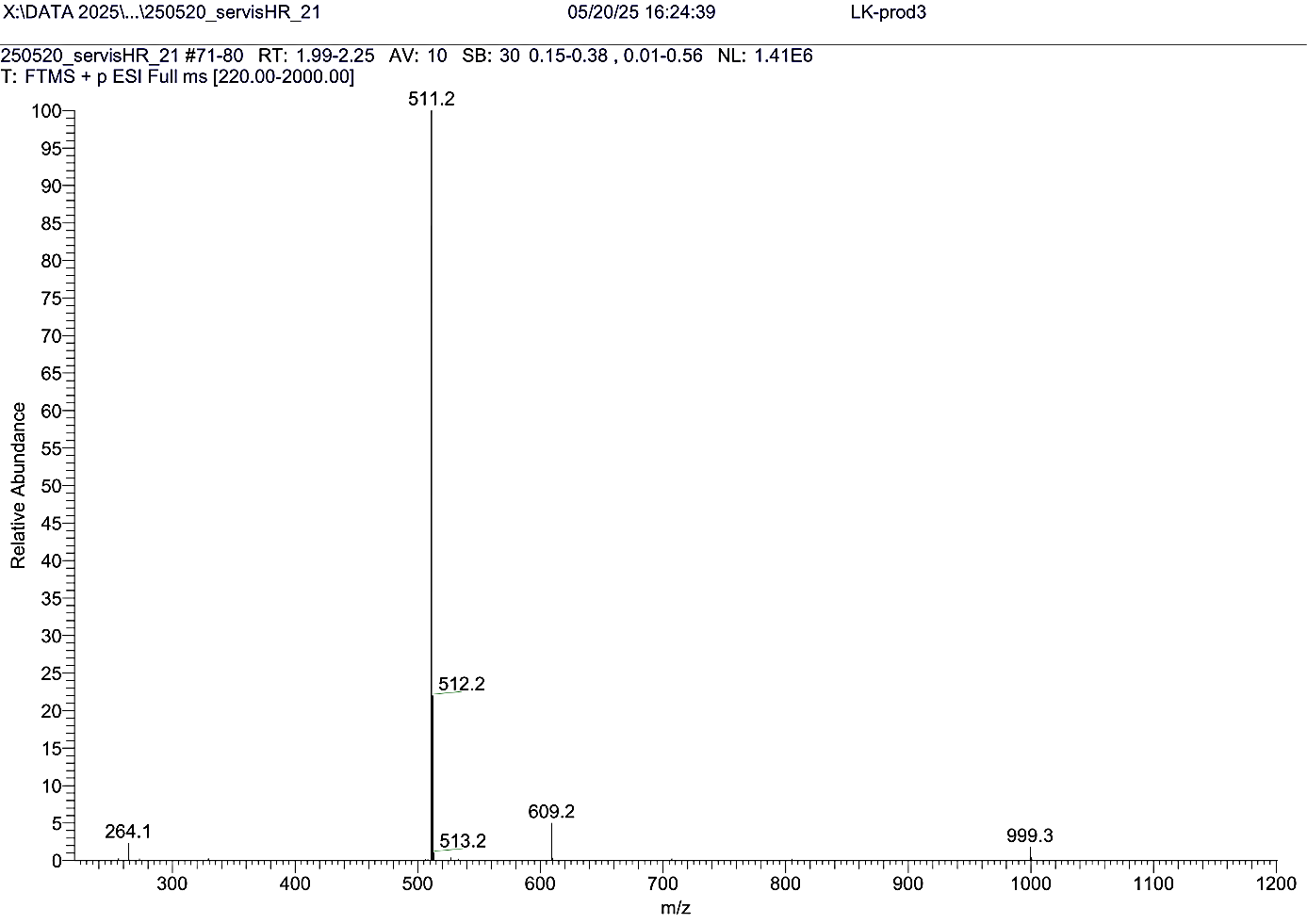


**Figure S8D.** MS spectrum (ESI^+^) of a mixture of compounds **7a** and **7b**: [M + Na]^+^, *m*/*z* 511.2. HRMS (ESI^+^): *m*/*z* for C_18_H_32_O_15_Na calculated 511.16334, found 511.16290 (-0.86794 ppm).
